# Tunable Transfer‐Hydrodeoxygenated Upgrading of Lignin‐Derived Propylphenols to Versatile Value‐Added Alkane‐Based Chemicals

**DOI:** 10.1002/advs.202500687

**Published:** 2025-03-11

**Authors:** Yanyan Yu, Yilin Li, Yuhan Lou, Mengyuan Chen, Yongzhuang Liu, Haipeng Yu

**Affiliations:** ^1^ Key Laboratory of Bio‐based Material Science and Technology of Ministry of Education State Key Laboratory of Utilization of Woody Oil Resource Northeast Forestry University Harbin 150040 P. R. China

**Keywords:** alkane‐based chemicals, combinational catalysts, hydrodeoxygenation, hydrogen‐transfer, lignin upgrading

## Abstract

Catalytic refining of lignin holds promise for producing sustainable platform chemicals. In this work, a gaseous hydrogen‐free catalytic hydrodeoxygenation system is developed for upgrading lignin‐derived phenols to alkane chemicals. Commercially available Raney Ni and HZSM‐5 are used as a combinational catalyst, with isopropanol serving as the hydrogen‐donating solvent. By modifying the temperature and the ratio of Raney Ni to HZSM‐5, the reaction pathways for hydrogenation and deoxygenation can be tailored to specific requirements. As a result, a 97.1% yield of alkane fuels is achieved, with 64.4% propylcyclohexane and 32.7% propylbenzene obtained in one‐pot reaction from the hydrodeoxygenation of 2‐methoxy‐4‐propylphenol using a 3:1 mass ratio of Ni to HZSM‐5, further increasing the ratio of HZSM‐5 leads to a selectively production of propylbenzene in 62.0% yield. Through careful regulation of the catalytic system and the design of hydrogenation–deoxygenation pathways, excellent yields of 4‐propylcyclohexanol (92.2%), propylcyclohexene (93.3%), and propylcyclohexane (93.2%) are directionally achieved. The catalyst maintained a conversion rate of over 99% after five cycles, demonstrating excellent robustness. This study offers a strategic system that expedites the selective upgrading of lignin‐derived chemicals, heralding a pathway toward sustainable fuels and chemicals.

## Graphical abstract

A gaseous hydrogen‐free catalytic system employing Raney Ni and HZSM‐5 with isopropanol upgrades lignin‐derived phenols into value‐added alkanes. By tuning catalyst ratios and temperature, selective pathways yield versatile products including propylbenzene (62%), 4‐propylcyclohexanol (92.2%), propylcyclohexene (93.3%), and propylcyclohexane (93.2%) with high efficiency. The robust system maintains >99% conversion over five cycles, enabling sustainable lignin valorization.

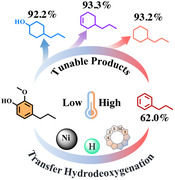

## 1 Introduction

Lignin, a complex and abundant natural polymer, is essential for the structural integrity and robustness of plants. As a plentiful renewable resource, it has been identified as a promising resource for the generation of bio‐based chemicals and materials.^[^ ^1^ ^]^ Recent research has vigorously explored diversiform depolymerization techniques, such as thermochemical processes, enzymatic treatments, and catalytic strategies.^[^ ^2^, ^3^, ^4^, ^5^, ^6^ ^]^ These efforts have successfully derived a variety of platform chemicals from lignin, including phenolic compounds, aromatic hydrocarbons, carboxylic acids, and lignin‐derived oils,^[^ ^7^, ^8^, ^9^, ^10^, ^11^ ^]^ presenting sustainable alternatives to petrochemical counterparts.^[^ ^12^, ^13^, ^14^ ^]^

Various methods are being developed to augment the utility of lignin monomer derivatives. Hydrodeoxygenation is one such promising approach, widely adopted for its efficacy in excising oxygen atoms from these derivatives.^[^ ^15^ ^]^ The process predominantly converts oxygen atoms into water, while concurrently reconfiguring the carbon structure of the lignin derivatives. This process generates a portfolio of valuable products, including aromatic compounds and aliphatic hydrocarbons that are useful as fuel sources.^[^ ^16^, ^17^, ^18^, ^19^, ^20^ ^]^ Precious metals like palladium (Pd) and ruthenium (Ru) are known for their superior catalytic efficacy in hydrodeoxygenation reactions.^[^ ^21^ ^]^ Zhao et al. utilized Pd/C to convert 2‐methoxy‐4‐propylphenol (1a) into 66% propylcyclohexane (1e) by introducing 5 MPa of hydrogen gas into a phosphoric acid solution with a pH of 2.1.^[^ ^22^ ^]^ In an effort to enhance the activity of monomeric lignin derivatives' hydrodeoxygenation for cycloalkane production, Luska et al. synthesized Ru nanoparticles immobilized on functionalized acidic ionic liquid phases (RuNPs@SILPs), and this approach effectively hydrodeoxygenated 2‐methoxy‐4‐methylphenol to yield 77% methylcyclohexane.^[^ ^23^ ^]^ Nevertheless, the process required high pressure (12 MPa) of gaseous hydrogen, and the yield of cycloalkanes decreased significantly with the increase of side chains. Due to cost considerations, the use of catalysts gradually shifted toward non‐precious metals. However, their efficiency in breaking C─O bonds remained inferior to that of precious metals. Numerous related studies have demonstrated that the synergistic use of an appropriate amount of acidic solid and redox metal catalyst significantly enhances the efficiency of hydrodeoxygenation of lignin derivatives.^[^ ^24^, ^25^, ^26^, ^27^, ^28^, ^29^, ^30^ ^]^

Solid acidic catalysts are essential in catalytic biomass deoxygenation due to their porous nature and the presence of numerous acidic sites.^[^ ^31^, ^32^ ^]^ Zeolites such as Hierarchical Zeolite Socony Mobil‐5 (HZSM‐5), Hydrogen‐type Y zeolite (H‐Y), Hydrogen‐type Beta zeolite (H‐β), Mobil Composition of Matter (MCM), and Ultra Stable Y zeolite (USY) are prominent examples of solid acids employed in tandem with metal catalysts to improve deoxygenation.^[^ ^33^ ^]^ The conversion and selectivity toward target products are significantly influenced by the distinct structural conformations and the specific acidic site characteristics inherent in each zeolite type. It is widely acknowledged that increasing temperature and hydrogen pressure can accelerate the hydrogenation and deoxygenation process, thereby improving the yield of the product.^[^ ^34^ ^]^ While the introduction of external molecular hydrogen effectively supplies the necessary hydrogen for both hydrogenation and deoxygenation steps, this approach brings substantial safety concerns, particularly the risks of hydrogen storage, transport, and operational handling. Therefore, there is a crucial demand for the development of an alternative hydrogenation and deoxygenation system, one that importantly operates without the need for molecular hydrogen, thereby mitigating the associated safety challenges.

The use of solvent‐based hydrogen sources was recently proposed to bypass the complications and hazards associated with external hydrogen gas supplies.^[^ ^35^ ^]^ Isopropanol, in particular, has been identified as an effective hydrogen‐donating solvent that can well minimize transport costs, reduce operational risks, and diminish the likelihood of equipment corrosion, etc.^[^ ^36^ ^]^ Additionally, non‐precious catalysts like Raney nickel (Ni) are capable of hydrogenating aromatic monomers into cyclohexanol at the comparatively low temperature of 140 °C when isopropanol is used as the solvent for hydrogen provision.^[^ ^37^ ^]^ In order to advance the industrialization of the conversion of lignin‐derived monomers into biofuels and platform chemicals, several key requirements must be fulfilled, including efficient deep hydrodeoxygenation, cost‐effectiveness, safe and convenient operation, as well as the use of non‐corrosive solvents for equipment. The hydrogen‐free catalytic system of Raney Ni combined with acidic zeolite using isopropanol as the hydrogen source demonstrates the potential to meet these challenges.^[^ ^38^, ^39^ ^]^ And, there is still a challenging issue of optimizing the effectiveness of these catalysts in deep hydrodeoxygenation within a gaseous hydrogen‐free environment.

**Scheme 1:**
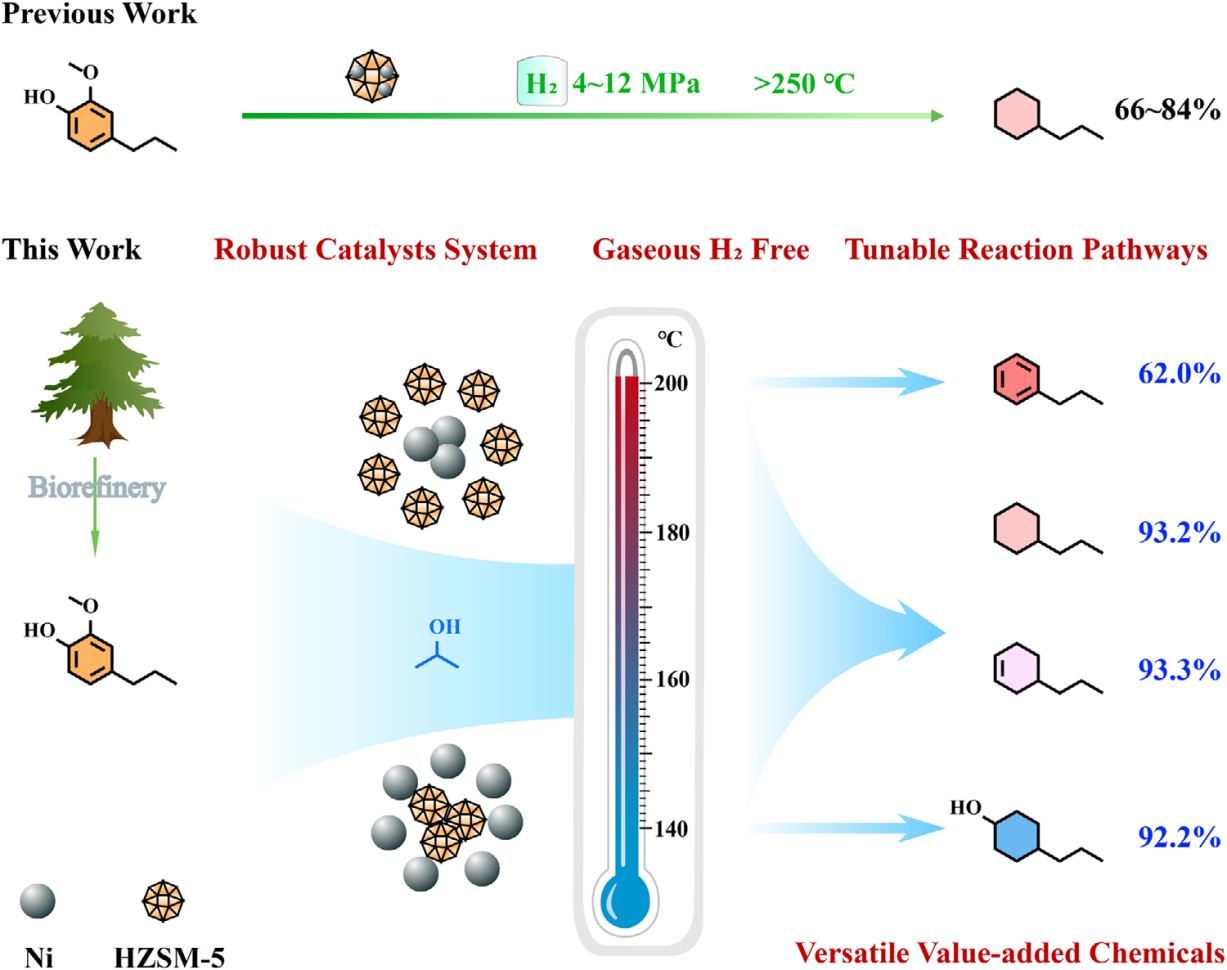
Schematic diagram shows the developed catalytic hydrogen‐transfer hydrodeoxygenation system and designed routes for upgrading lignin‐derived phenols to value‐added chemicals.

This research presents a detailed investigation into the tailored, hydrogen‐free upgrading of lignin‐derived monomers utilizing Raney Ni&HZSM‐5 combinational catalysts. The primary objective was to assess the efficacy and selectivity of the catalysts for the targeted conversion of lignin‐derived monomers into alkylalcohols, alkylolefins, cycloalkanes, and alkylbenzenes (**Scheme** **1**). We systematically examined variables such as catalyst composition, reaction temperature, residence time, the ratio of metal catalysts to zeolite and dosage of hydrogen‐donating solvent to refine conversion rates and product specificity. Additionally, the research delved into the effects of catalyst characteristics, including Ni content and zeolite acidity, on overall catalytic behavior and catalyst durability. Gas chromatography (GC) was used to facilitate the identification and quantification of various compounds generated during the upgrading process, providing insights into the reaction pathways and mechanisms involved. This work would provide theoretical guidance for the directional conversion of lignin derivatives into alkane fuels and aromatic chemicals, contributing to the upgrading of sustainable low‐value feedstocks to high‐value chemicals.

## 2 Results and Discussion

### 2.1 Screening the Transfer‐Hydrogenation Systems

To achieve cost‐effective hydrogenation of 2‐methoxy‐4‐propylphenol, we employed a Raney Ni catalyst to mediate the hydrogen transfer reaction under moderate conditions, thereby obviating the need for external hydrogen sources. Isopropanol was utilized as a hydrogen‐donating solvent, supplying the requisite hydrogen for the hydrogenation of 2‐methoxy‐4‐propylphenol at 140 °C. To evaluate the efficiency of hydrogen transfer, comparative analyses were conducted on three alternative metal catalysts (Ru/C, Pd/C, and Ni/SiO_2_‐Al_2_O_3_) that were previously reported to exhibit proficient hydrogenation.^[^ ^40^, ^41^ ^]^ Concurrently, the efficiency of various alcohols (glycerol, ethylene glycol, ethanol, and methanol) alongside water as hydrogen donors was also scrutinized.^[^ ^42^, ^43^ ^]^

From the conversion rate of 2‐methoxy‐4‐propylphenol in **Table** **1**, it can be seen that compared to other catalysts, Raney Ni has better transfer hydrogenation performance in isopropanol solution. Further characterization of the catalytic activity of each catalyst shows that Ru/C has the highest turnover frequency (TOF) value, but the selectivity for 4‐propylcyclohexanol (1b) is not high. In contrast, Raney Ni demonstrates superior catalytic activity, achieving a high hydrogenation conversion rate for 2‐methoxy‐4‐propylphenol and selectivity of 82.6% for 4‐propylcyclohexanol. The conversion efficiency of the Ni/SiO_2_‐Al_2_O_3_ catalyst, which contains metallic Ni, is comparatively low. This reduced efficiency may be attributed to an oxide layer on the Ni surface, which obstructs the interaction between metallic Ni and the substrate, thereby hindering Ni's catalytic hydrogenation. At the same time, based on the results of Raney Ni catalysis alone and Raney Ni/HZSM‐5 mixed catalysis in Table 1, the addition of SiO_2_‐Al_2_O_3_ will hinder the hydrogenolysis and hydrogenation reactions of 2‐methoxy‐4‐propylphenol. From the gradually increasing propylcyclohexane in Figure S1 (Supporting Information), it can be seen that Raney Ni is activated during the heating process to catalyze the 4‐propylcyclohexanol hydrogenolysis to propylcyclohexane, indicating the importance of Raney Ni in the hydrogenolysis and hydrogenation processes.

**Table 1:** Different hydrogenation reaction systems and corresponding conversion of 2‐methoxy‐4‐propylphenol and yield of 4‐propylcyclohexanol.

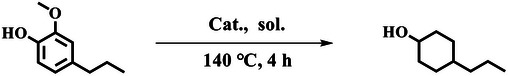				
Catalyst	Solvent	Conv. of 1a%	Select of 1b%	TOF(%/h)
^a)^Ru/C	Isopropanol	30.5	42.8	134.0
^a)^Pd/C	Isopropanol	0.5	–	2.7
^a)^Ni/SiO_2_‐Al_2_O_3_	Isopropanol	1.6	–	0.4
^b)^Raney Ni	Isopropanol	99.87	82.7	29.3
^b)^Raney Ni	Glycerol	3.8	–	1.1
^b)^Raney Ni	Ethylene glycol	13.5	–	3.3
^b)^Raney Ni	Ethanol	3.4	–	0.8
^b)^Raney Ni	Methanol	2.5	–	0.6
^b)^Raney Ni	Water	40.9	17.1	10.0
^c)^Raney Ni	Isopropanol	93.8	82.6	110.1
^c)^Raney Ni/HZSM‐5	Isopropanol	76.3	60.8	89.5

^a)^ Reaction condition: 2 mmol 2‐methoxy‐4‐propylphenol, 0.2 g catalyst (for Ru/C and Pd/C, the metal load is 5 wt.%; while for Ni/SiO_2_‐Al_2_O_3_, the metal Ni load is 65 wt.%), 10 mL isopropanol, 140 °C, 4 h;

^b)^ Reaction condition: 2 mmol 2‐methoxy‐4‐propylphenol, 0.5 g Raney Ni, (wet, water content 80 wt.%), 10 mL solvent, 140 °C, 4 h;

^c)^ Reaction condition: 2 mmol 2‐methoxy‐4‐propylphenol, 1 g Raney Ni (wet, water content 80 wt.%), 0 g or 0.25 g HZSM‐5, 10 mL isopropanol, 140 °C, 0.5 h;

The TOF value is calculated by dividing the number of moles of metal catalyzed substrate conversion per unit mole by the reaction time (h). Multiply by the percentage and display the activity of different catalysts more clearly in units of %/h.

**Figure 1:**
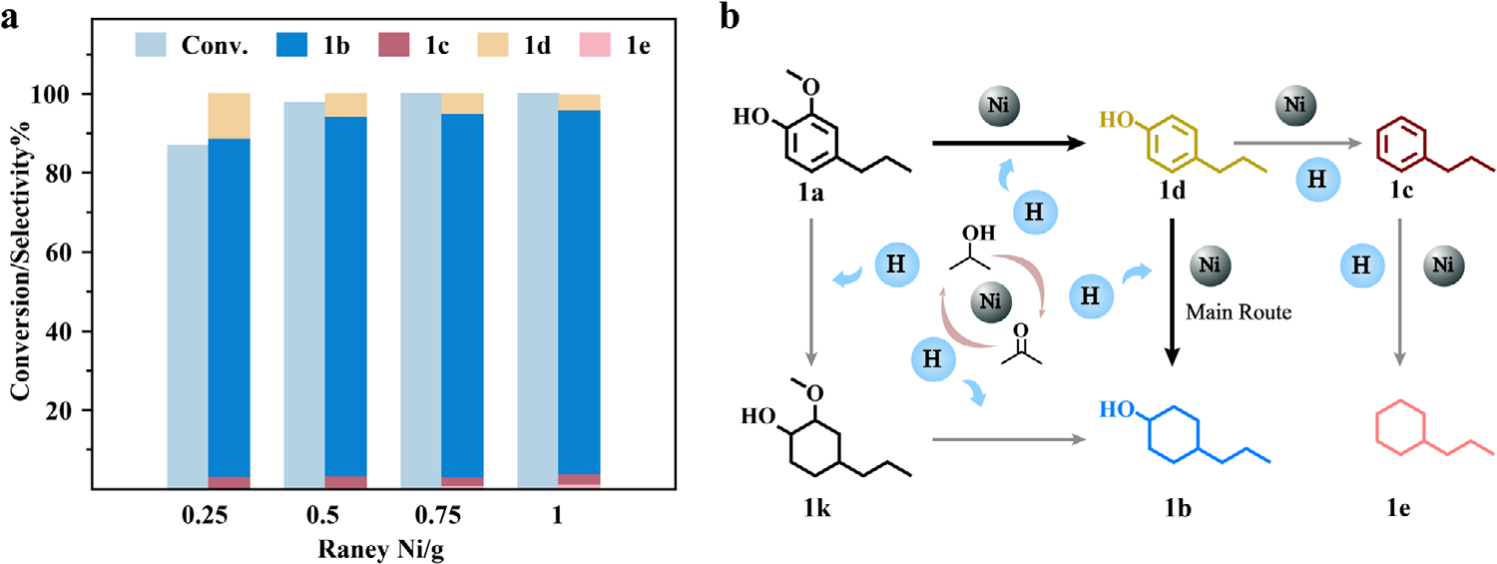
a) Effect of Raney Ni dosage on 2‐methoxy‐4‐propylphenol conversion, b) Pathway for the conversion of 2‐methoxy‐4‐propylphenol (1a) to 4‐propylcyclohexanol (1b), propylbenzene (1c), 4‐propylphenol (1d), propylcyclohexane (1e), and 2‐methoxy‐4‐propylcyclohexane (1k). Reaction condition: 2 mmol 2‐methoxy‐4‐propylphenol, 0.25–1.0 g Raney Ni, 10 mL isopropanol, 140 °C, 4 h.

Upon ascertaining the most effective hydrogenation system for 2‐methoxy‐4‐propylphenol, we proceeded to fine‐tune the reaction by modifying the concentration of Raney Ni. An enhancement in hydrogen production capability correlated positively with the increased amount of Raney Ni (**Figure** **1**a). This augmentation led to a complete conversion of 2‐methoxy‐4‐propylphenol. The product profile was predominated by 4‐propylcyclohexanol at a selectivity of 92.2%, accompanied by minor proportions of propylbenzene (1c), 4‐propylphenol (1d), and propylcyclohexane.

The inferred hydrogenation pathway for substrate 2‐methoxy‐4‐propylphenol has been elucidated on the basis of product distribution and mechanistic investigations (Figure 1b). Upon temperature induction, the Raney Ni catalyst experiences gradual activation, facilitating the binding of hydrogen to the hydroxyl group of isopropanol. Isopropanol serves as the primary hydrogen donor in the initial hydrogenation of 2‐methoxy‐4‐propylphenol to 4‐propylcyclohexanol. Upon its adsorption at the active Ni sites, isopropanol undergoes cleavage of the C─H bond adjacent to the hydroxyl group, resulting in the formation of acetone and H₂.^[^ ^23^, ^37^ ^]^ The released H₂ dissociates, forming hydrogen atoms on the Ni surface that are crucial for facilitating both the hydrogenolysis and intra‐cyclic hydrogenation of 2‐methoxy‐4‐propylphenol. This process also enables the subsequent hydrogenation of 4‐propylphenol to 4‐propylcyclohexanol. Consequently, the hydrogenation reaction within this system can be performed without the need for external hydrogen gas. The results indicate that the isopropanol‐mediated hydrogen transfer, facilitated by Raney Ni, effectively promotes the preliminary hydrogenolysis and hydrogenation of 2‐methoxy‐4‐propylphenol under moderate conditions.^[^ ^38^ ^]^ However, to achieve the desired transformation into a cycloalkane, further advancements in the deoxygenation reaction are necessary.

### 2.2 One‐Pot Hydrodeoxygenation Reaction and Products Distribution

**Figure 2:**
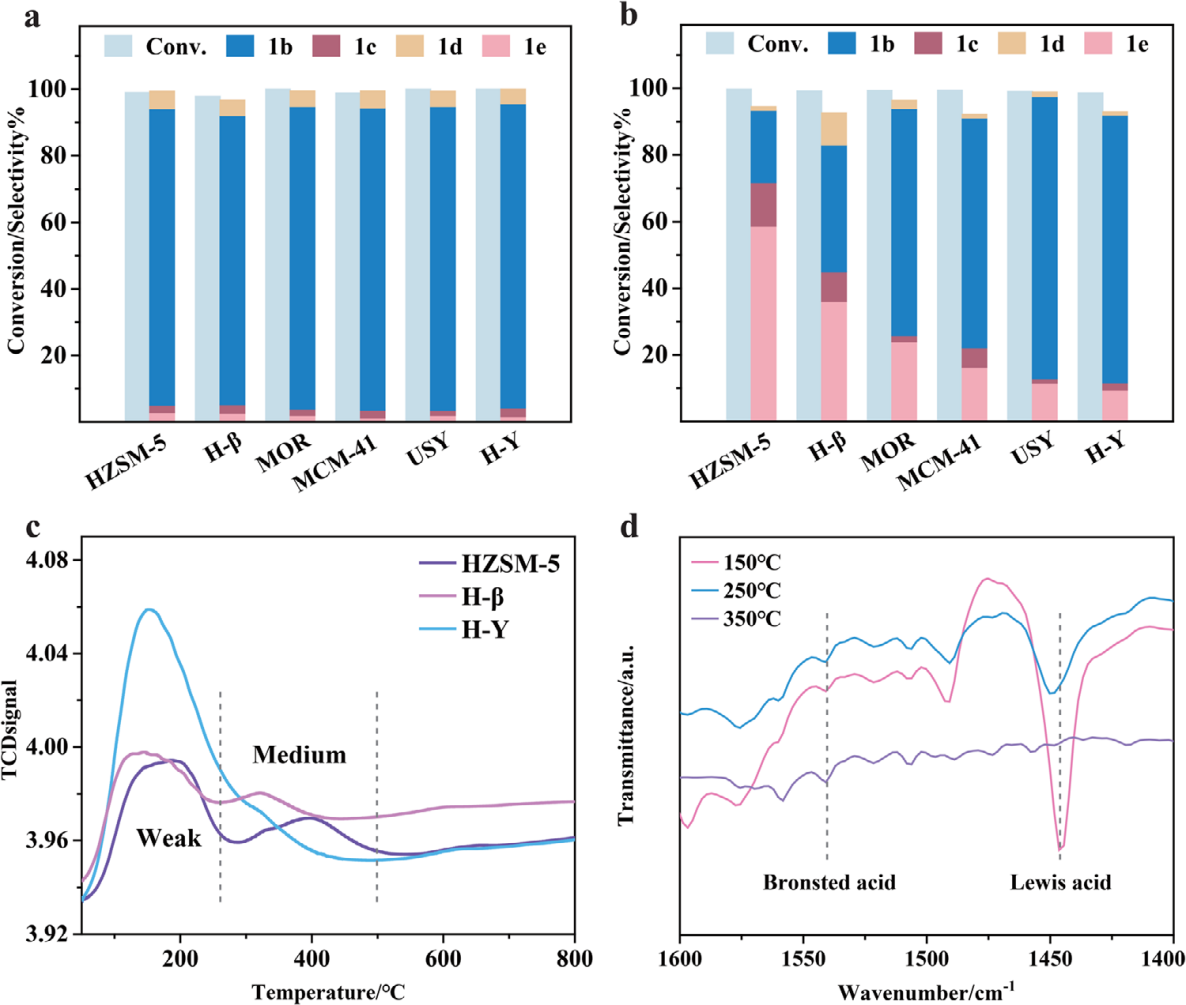
Hydrodeoxygenation reactions after zeolite addition. a) Substrate conversion and product distribution after reaction at 140 °C. b) Substrate conversion and product distribution after reaction at 200 °C. c) Ammonia temperature‐programmed desorption (NH_3_‐TPD) diagrams of three types of zeolite HZSM‐5, H‐β, and H‐Y. d) Pyridine adsorption Fourier‐transform infrared (Py‐IR) of combinational catalyst Raney Ni&HZSM‐5. Reaction condition: 2 mmol substrate of 2‐methoxy‐4‐propylphenol, 1 g Raney Ni, 0.25 g zeolite, 10 mL isopropanol, 4 h. Abbreviations and full names in the picture: 4‐propylcyclohexanol (1b), propylbenzene (1c), 4‐propylphenol (1d), propylcyclohexane (1e).

During the Raney Ni‐catalyzed hydrogenation process, 4‐propylcyclohexanol is predominantly produced as the deoxygenation ability of the catalyst is confined to the cleavage of the ether bond in the methoxy group. To increase the yield of the targeted deoxygenation product, propylcyclohexane, in one pot, it is necessary to integrate an acidic catalyst in a calibrated amount. The selected zeolite delivers a complementary effect in combination with Raney Ni. The inclusion of six commercially available zeolites (HZSM‐5, H‐Y, H‐β, USY, MOR, and MCM‐41) into the refined hydrogenation system aims to steer a one‐pot reaction toward the production of cycloalkane. It is discerned that the reaction temperature is a critical factor influencing the deoxygenation of 4‐propylcyclohexanol. At lower temperatures, specifically 140 °C, the zeolites' reactivity is not induced, as shown in **Figure** **2**a, resulting in a product distribution that mirrors the outcomes in the absence of zeolite catalysts.

To activate the activity of the zeolites' acidic sites, the reaction temperature is incrementally elevated, which in turn, enhances the yield of the deoxygenated product, propylcyclohexane (Figure S2, Supporting Information). Concurrently, the reaction pressure is progressively increased, with a temperature ceiling of 200 °C being maintained to prioritize operational safety. Alongside, the product distribution in the absence of zeolites is scrutinized as the temperature is systematically increased to 200 °C, underscoring the pivotal contribution of zeolites to the hydrodeoxygenation process.

Furthermore, at an elevated temperature of 200 °C, the deoxygenation efficiency of different zeolites significantly improves, with HZSM‐5 outperforming others by achieving the highest rates of deoxygenation, followed by the H‐β zeolite, as shown in Figure 2b. In contrast, the H‐Y zeolite demonstrates the lowest deoxygenation efficiency. By examining the adsorption and desorption performance of these three zeolites through NH_3_‐TPD (Figure 2c), we explore the reasons for the excellent deoxygenation effect of HZSM‐5 from the perspective of acid sites. It is found that a significant amount of desorption occurs on the surface of zeolites only when the temperature reaches 200 °C. At ≈200 °C, it is mainly the desorption of weak acid sites, while at ≈400 °C, it is the desorption of medium strong acid sites. This indicates that there are more medium strong acid active centers distributed in the HZSM‐5 zeolite.^[^ ^44^, ^45^ ^]^ The Py‐FTIR test results of Ni&HZSM‐5 (Figure 2d) show that the zeolite contained a higher proportion of Lewis acid sites. As the temperature increased (150–350 °C), the Lewis acid site content significantly decreased, indicating that the main catalytic acid site type in the reaction system at 200 °C was Lewis acid site.

The findings indicate that HZSM‐5 contains more medium strong acid sites, is beneficial for the deoxygenation reaction.^[^ ^46^ ^]^ And the NH_3_ desorption peak of weak acids shifts to the right at 200 °C. Therefore, under this reaction system and temperature conditions, HZSM‐5 is the best choice compared to other zeolites. Conversely, although the H‐Y zeolite demonstrates a higher total acidity (Table S1, Supporting Information), it primarily consists of weak acids, which are inadequate for the effective removal of the hydroxyl group from 4‐propylcyclohexanol.^[^ ^47^ ^]^

When the temperature reaches 200 °C, the hydrogen adsorbed on the zeolite begins to desorb, gradually exposing acidic sites. Under the synergistic effect of Lewis and Brønsted acids, the hydroxyl groups in 4‐propylcyclohexanol are broken, producing more propylcyclohexane. This demonstrates that the Raney Ni&HZSM‐5 catalyst is qualified to realize one‐pot high‐efficiency conversion of 2‐methoxy‐4‐propylphenol to cycloalkane. At the same time, we noticed that the outer layer of the catalyst appeared black during the reaction at 140 °C and white at 200 °C (Figure S3, Supporting Information), which proves the previous statement that Raney Ni and HZSM‐5 can change catalytic sites at 140 and 200 °C.

### 2.3 Optimization of Reaction Pathways

**Figure 3:**
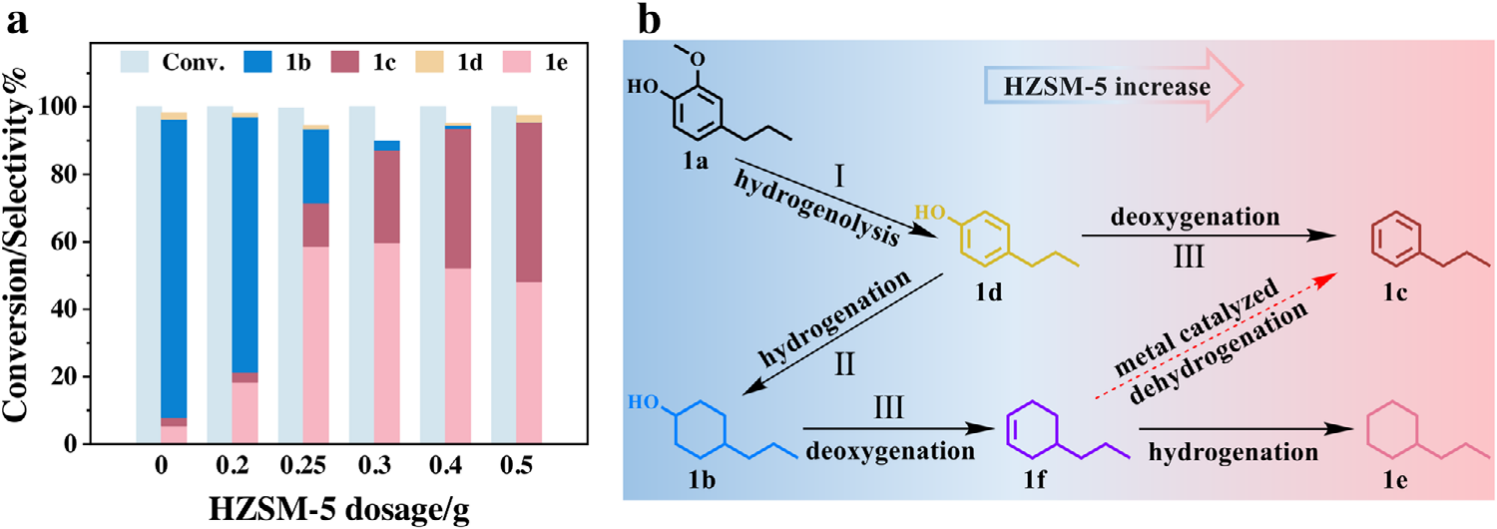
a) Optimization of product distribution for yielding cyclohexane. b) Reaction pathways of hydrodeoxygenation. Reaction condition: 2 mmol 1a (2‐methoxy‐4‐propylphenol), 1 g Raney Ni, 0–0.5 g HZSM‐5, 10 mL isopropanol, 200 °C, 4 h. Abbreviations and full names in the picture: 4‐propylcyclohexanol (1b), propylbenzene (1c), 4‐propylphenol (1d), propylcyclohexane (1e), propylcyclohexene (1f).

Through the above optimizations, we have designed a physically blended Raney Ni&HZSM‐5 catalyst to efficiently convert 2‐methoxy‐4‐propylphenol into alkylbenzenes and cycloalkanes in a one‐pot reaction. To achieve a better cycloalkane yield, it is necessary to further optimize the dosage and ratio of Raney Ni to HZSM‐5. **Figure** **3**a depicts the correlations between varying HZSM‐5 dosages, conversion rates, and product distribution. An initial increase in the yield of propylcyclohexane occurs with rising quantities of the zeolite, which then subsequently exhibits a tapering decline. Conversely, the production of 4‐propylcyclohexanol consistently decreases. These trends suggest that escalating HZSM‐5 concentrations facilitate the removal of the alcohol hydroxyl group from 4‐propylcyclohexanol, prompting its transformation into propylcyclohexane. Nevertheless, beyond a certain threshold of HZSM‐5 addition, the yield of propylcyclohexane does not further increase but instead demonstrates a slight downward tendency. Instead, another interesting compound, propylbenzene, was produced with high yield and selectivity, highlighting its importance.^[^ ^48^ ^]^ Empirical evidence indicates that the yield of propylbenzene is intricately linked to the concentration and acidic site density of the zeolite. Correspondingly, as the zeolite dosage is augmented, there is a concurrent increase in reaction pressure. Due to safety considerations, we chose to cap the HZSM‐5 dosage at 0.5 g.

The product distribution data delineate three main pathways in the hydrogenation and deoxygenation process, as depicted in Figure 3b. The data suggest a preferential conversion pathway during the reaction process. Initially, pathway I is predominant, where metallic Ni catalyzed hydrogenolysis of 2‐methoxy‐4‐propylphenol, yielding modest quantities of compound 4‐propylphenol.^[^ ^49^ ^]^ As the availability of the hydrogen source increases, pathway II takes precedence, leading to the hydrogenation of the ring and concomitant production of compound 4‐propylcyclohexanol. Concurrently, an elevation in reaction temperature progressively activates the acidic sites of the zeolites, thus triggering the deoxygenation reaction (pathway III) of 4‐propylcyclohexanol, is converted to propylcyclohexene (1f), which in turn is rapidly converted to propylcyclohexane in the presence of a sufficient hydrogen source.^[^ ^50^ ^]^ This results in the gradual consumption of 4‐propylcyclohexanol to form propylcyclohexane through the combined pathways of hydrogenation and deoxygenation (II+III). With an excess of acidic zeolites, after the deoxygenation of 4‐propylcyclohexanol is complete, the surplus zeolite fosters an acidic environment conducive to the dehydrogenation reaction,^[^ ^51^ ^]^ the presence of metals and acidic environment suggests a potential for dehydrogenation of olefin product, manifesting in an incremental formation of the propylbenzene product.

**Figure 4:**
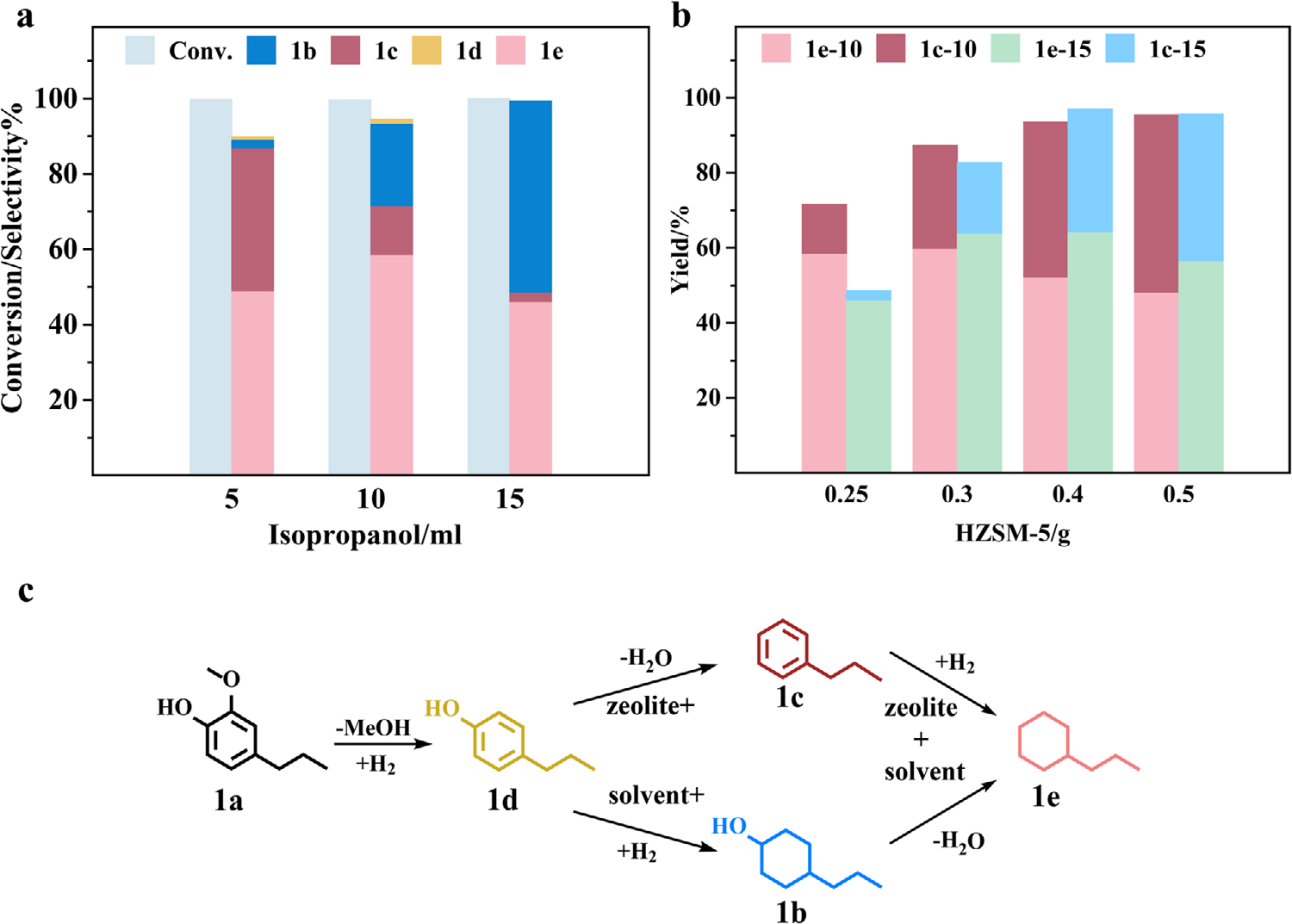
a) Conversion and product distribution under different isopropanol volume (5, 10, 15 mL) and 0.25 g HZSM‐5. b) The yield of propylcyclohexane and propylbenzene under different HZSM‐5 dosage (0.25–0.5 g) and isopropanol volumes (10, 15 mL). c) propylcyclohexane production pathway. Reaction condition: 2 mmol 2‐methoxy‐4‐propylphenol (1a), 1 g Raney Ni, 200 °C, 4 h. Abbreviations and full names in the picture: 4‐propylcyclohexanol (1b), propylbenzene (1c), 4‐propylphenol (1d), propylcyclohexane (1e).

Our investigation indicates that the inability to increase the yield of propylcyclohexane may be linked to an excess of HZSM‐5 used in the concurrent catalytic processes of hydrogenation and deoxygenation. This surplus appears to skew the reaction toward prevalent deoxygenation, culminating in a higher production of compound propylbenzene. We posit that the enhancement of the yield of propylcyclohexane is potentially hindered by an inadequate supply of hydrogen originating from isopropanol. Empirical modifications of isopropanol levels demonstrate that augmenting its concentration fails to substantially raise the yield of propylcyclohexane (**Figure** **4**a). Notably, we observe a concurrent decrease in the yield of propylbenzene alongside an increment in 4‐propylcyclohexanol generation, suggesting that a sufficient hydrogen source facilitates the hydrogenation process, driving the conversion of benzene derivatives to cycloalkanol. The inference is that severing the hydroxyl groups on cycloalkanol is vital for amplifying the yield of propylcyclohexane.

Bearing in mind the deoxygenating influence of the zeolite, we experimented with increasing the zeolite quantity to more effectively transmute 4‐propylcyclohexanol into propylcyclohexane. This modification resulted in a slight elevation in the yield of propylcyclohexane while predominantly converting 4‐propylcyclohexanol into propylbenzene. In order to further clearly reveal the synergistic role of HZSM‐5 and hydrogen‐donating solvents in the production of propylcyclohexane and propylbenzene by hydrodeoxygenation, we have scrutinized the yield changes of propylcyclohexane and propylbenzene with increased solvent volume at different zeolite dosages. As illustrated in Figure 4b, the yield of propylcyclohexane increases and then decreases after increasing the amount of zeolite, reaching a peak yield at a dose of 0.3 g of HZSM‐5 zeolite. Beyond a zeolite threshold of 0.3 g, elevating the isopropanol solvent levels can further enhance the yield of propylcyclohexane and decrease the yield of propylbenzene.

Examination of the product evolution pathway indicates that a higher concentration of zeolite catalyzes deoxygenation, thereby accelerating the conversion of 4‐propylphenol to propylbenzene, which results in a progressive increase in the yield of propylbenzene (Figure 4c). Simultaneously, this deoxygenation preference limits the hydrogenation of 4‐propylphenol to 4‐propylcyclohexanol, diminishing the formation of propylcyclohexane. However, the addition of a hydrogen donor solvent in an optimal range (10–15 mL) can enhance the hydrogenation process of propylbenzene to propylcyclohexane, leading to an improved yield of propylcyclohexane. Therefore, the optimal conditions to obtain the highest yield of propylcyclohexane with guaranteed yield and cost savings are: adding 0.3 g of HZSM‐5 in 15 mL of isopropanol solution and reacting at 200 °C for 4 h. On the other hand, 0.5 g of HZSM‐5 and 10 mL of isopropanol are added to obtain a higher yield of propylbenzene product.

**Figure 5:**
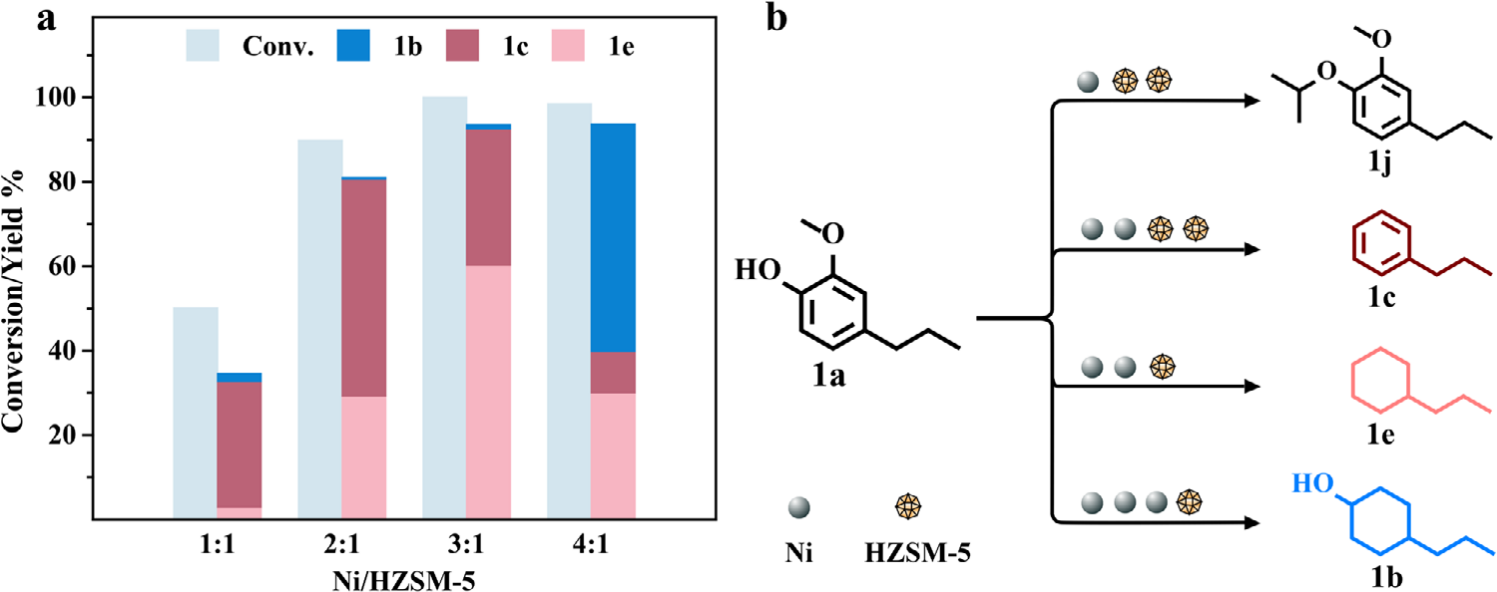
a) Conversion and product distribution at different ratios of Ni to HZSM‐5. b) Conversion path at different ratios of Ni to HZSM‐5. Reaction condition: 2 mmol 2‐methoxy‐4‐propylphenol (1a), 0.3–1.2 g Raney Ni, 0.3 g zeolite, 10 mL isopropanol, 200 °C, 4 h. Abbreviations and full names in the graph: 2‐methoxy‐4‐propylphenol (1a), 4‐propylcyclohexanol (1b), propylbenzene (1c), propylcyclohexane (1e), 1‐isopropoxy‐2‐methoxy‐4‐propylbenzene (1j).

The hydrodeoxygenation process is mediated by the cooperative interactions between metal and solid acid catalyst. An investigation into the impact of the Raney Ni to HZSM‐5 ratio on product distribution is therefore pivotal (**Figure** **5**a). It was observed that as the Raney Ni to HZSM‐5 ratio increased, the conversion rate of 2‐methoxy‐4‐propylphenol also increased. At reduced Raney Ni concentrations, deoxygenation reactions, primarily driven by the zeolite catalysts, were predominant. The Raney Ni synergistically interacted with HZSM‐5 to facilitate the removal of methoxy and phenolic hydroxyl groups. Furthermore, some isopropanol was attached to the phenolic hydroxyl groups of 2‐methoxy‐4‐propylphenol, catalyzed by the acidic zeolites, a process evidenced by the products appearing between 13–15 min on GC peak positions (Figure S4, Supporting Information). Conversely, intracyclic hydrogenation was less favored competitively, yielding a reduced formation of propylcyclohexane. However, an initial increase followed by a decrease in the yield of propylcyclohexane was noted with higher Raney Ni proportions, concurrent with an increase in 4‐propylcyclohexanol production, indicating resurgence in the dominance of Raney Ni‐catalyzed hydrogenation and isopropanol transfer hydrogenation.

As depicted in Figure 5b, an excess of HZSM‐5 leads to the formation of the grafted product 1‐isopropoxy‐2‐methoxy‐4‐propylbenzene (1j), whereas an optimal HZSM‐5 concentration results in the enhanced production of propylbenzene. To this end, exceeding the Raney Ni to HZSM‐5 ratio of 3:1 results in a decreased yield of propylbenzene and an increase in the yield of propylcyclohexane. By combining the optimized conditions of HZSM‐5 and isopropanol, it can be seen that the highest yield of alkane fuel (97.1%, contains 64.4% of propylcyclohexane and 32.7% of propylbenzene) will be obtained when 15 mL of isopropanol is added during the hydrodeoxygenation conversion of 2‐methoxy‐4‐propylphenol, if the mass ratio of Raney Ni to HZSM‐5 is kept at 3:1. The change in the yield of propylbenzene is more closely related to that of the acidic zeolite; thus, the highest yield of propylbenzene (47.2%) is obtained when the mass ratio of Raney Ni to HZSM‐5 changed into 2:1 and the volume of isopropanol is decreased to 10 mL.

### 2.4 Reaction Mechanism of the Hydrodeoxygenation Process

**Figure 6:**
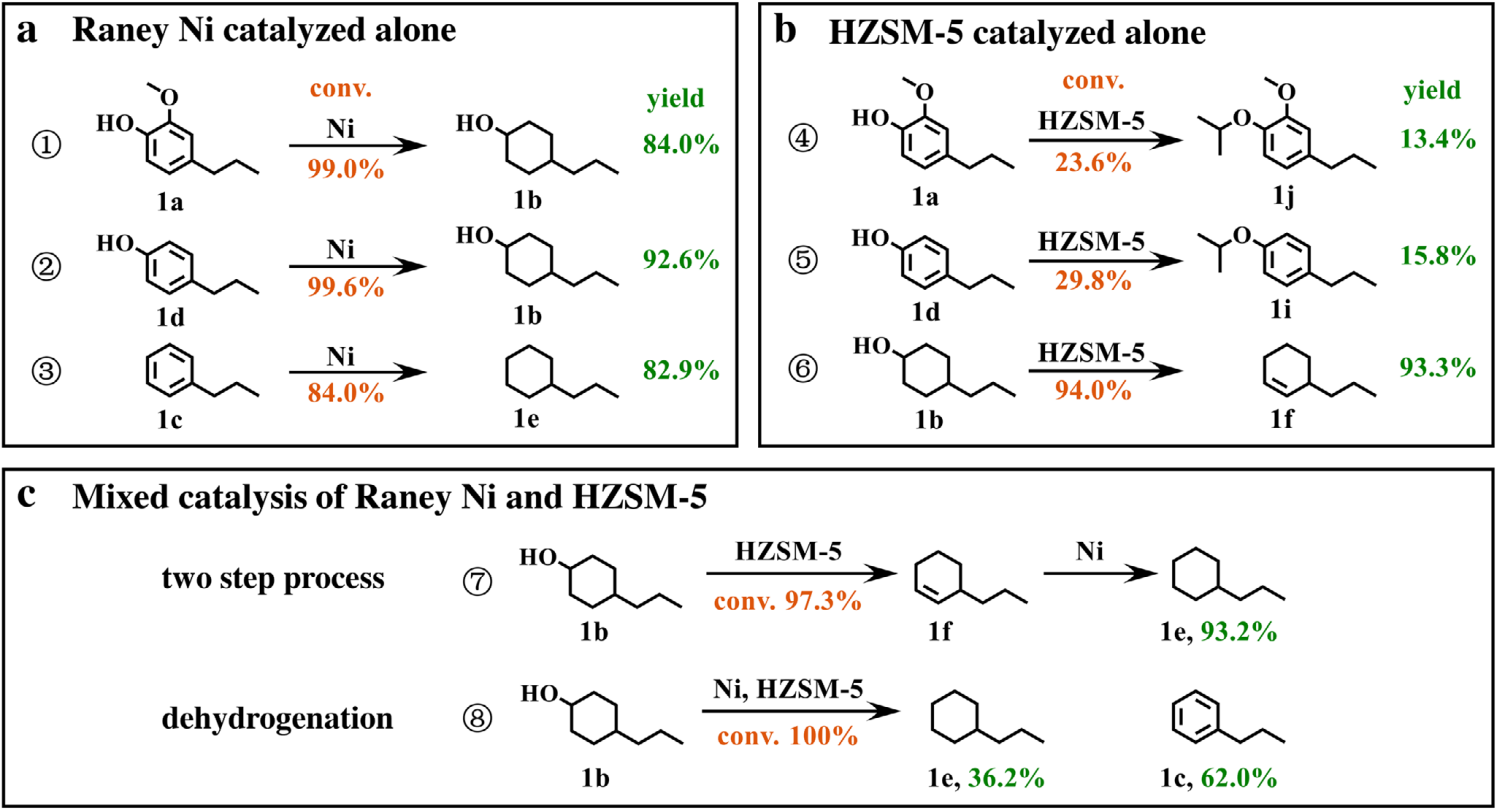
Validation of the separate roles of Raney Ni and HZSM‐5 in the hydrodeoxygenation reaction pathway. a) Conversion and yield of Raney Ni (1 g) catalyzed hydrogenolysis and hydrogenation reactions of 2‐methoxy‐4‐propylphenol and its intermediates, respectively. b) Conversion and yield of HZSM‐5 (0.25 g) catalyzed deoxygenation and alkoxylation reaction of 2‐methoxy‐4‐propylphenol and its intermediates, respectively. c) Path validation of 4‐propylcyclohexanol in the hydrodeoxygenation and dehydrogenation process, **⑦**: Raney Ni (1 g) and HZSM‐5 (0.25 g) catalysts were added in two separate steps, **⑧**: One‐step addition of Raney Ni (0.5 g) and HZSM‐5 (0.3 g) to verify the dehydrogenation pathway for the conversion of 4‐propylcyclohexanol to propylbenzene. Reaction condition: 2 mmol substrate, 10 mL isopropanol, 200 °C, 4 h. Abbreviations and full names in the picture: 2‐methoxy‐4‐propylphenol (1a), 4‐propylcyclohexanol (1b), propylbenzene (1c), 4‐propylphenol (1d), propylcyclohexane (1e), propylcyclohexene (1f), 4‐propanoxypropylbenzene (1i), 1‐isopropoxy‐2‐methoxy‐4‐propylbenzene (1j).

To rigorously assess the individual contributions of the metal catalyst and zeolite to the hydrogenation and deoxygenation reactions within this system, individual experiments were conducted. GC results for the varied substrates are presented in Figure S5 (Supporting Information) to facilitate a more precise comparative analysis. Under the catalysis of Raney Ni alone, with 2‐methoxy‐4‐propylphenol and its intermediate products propylbenzene and 4‐propylphenol as substrates, the metal catalyst is able to break the methoxy group on the side chain and hydrogenate the benzene ring of 2‐methoxy‐4‐propylphenol with the participation of isopropanol to produce 4‐propylcyclohexanol (reactions **①②③** in **Figure** **6**a). Comparing the yields of 4‐propylcyclohexanol in reaction **②** and propylbenzene of reaction **③** indicates that the presence of hydroxyl groups in the aromatic monomer is favorable for its hydrogenation (see Figure S6, Supporting Information).

As shown in reactions **④** and **⑤** in Figure 6b, in the deoxygenation reaction involving only the HZSM‐5 acid catalyst, the conversions of both aromatic monomers 2‐methoxy‐4‐propylphenol and 4‐propylphenol are very low (23.6% and 29.8%, respectively), and it is not possible to break the C─O bond on the monomers. Instead, the solvent isopropanol is grafted onto the monomers through an acid‐catalyzed dehydration condensation reaction of the phenolic hydroxyl group with the alcohol hydroxyl group to form the 1‐isopropoxy‐2‐methoxy‐4‐propylbenzene (1j) and 4‐propanoxypropylbenzene (1i) products. However, 4‐propylcyclohexanol, which has been initially hydrogenated, can be catalytically converted by HZSM‐5 to form the deoxygenated product propylcyclohexene with a high conversion of 93.3% (reaction **⑥** in Figure 6b). Detailed GC results for reactions **④**, **⑤**, and **⑥** are shown in Figure S7 (Supporting Information). We thus confirm the unique utility of the metal catalyst Raney Ni for breaking methoxy groups and catalyzing the hydrogenation of benzene rings in the hydrotransformation of aromatic monomers. In contrast, the acid catalyst HZSM‐5 shows significant differences in its deoxygenation effect against different types of hydroxyl groups, being more adept at removing alcohol hydroxyl groups on cycloalkanols and unable to disconnect phenol hydroxyl groups.

In the successive reactions of hydrogenation and deoxygenation of 2‐methoxy‐4‐propylphenol, the more abundant product obtained is propylcyclohexane, while the yield of propylcyclohexene is extremely low. Therefore, after the deoxygenation of 4‐propylcyclohexanol to form propylcyclohexene, it undergoes another step of hydrogenation. This two‐step reaction using 4‐propylcyclohexanol as the substrate confirms this speculated pathway (reaction **⑦** in Figure 6c; see Figure S8, Supporting Information). It is further clarified that Raney Ni dominates the hydrogenation reaction, and HZSM‐5 dominates the deoxygenation of cycloalkanols. During the validation of these two types of catalytic reactions separately, it is found that neither the metal catalyst nor the acid catalyst has a good breaking effect on the phenolic hydroxyl group. However, in the hydrodeoxygenation sequential reaction, by adjusting the ratio of HZSM‐5, we can again obtain propylbenzene in 47.2% yield, and therefore, the validation of the pathway for the production of propylbenzene is very necessary. In reaction **⑧** in Figure 6c, when propylcyclohexanol is used as the substrate and both Raney Ni and HZSM‐5 are added, a yield of propylbenzene as high as 62.0% is achieved, which confirms the existence of the metal‐acid co‐catalyzed dehydrogenation pathway^[^ ^51^ ^]^ as illustrated in Figure 3b.

### 2.5 Reaction Kinetics of the Hydrodeoxygenation Process

**Figure 7:**
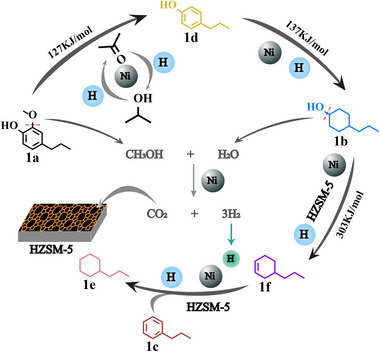
Possible pathways for the hydrogenation and deoxygenation conversion of substrate 2‐methoxy‐4‐propylphenol (1a) to propylcyclohexane (1e) and propylbenzene (1c). Abbreviations and full names in the picture: 4‐propylcyclohexanol (1b), 4‐propylphenol (1d), propylcyclohexene (1f).

The overall analysis of the hydrodeoxygenation pathway of compound 2‐methoxy‐4‐propylphenol through a combination of hydrogenolysis, hydrogenation, and hydrodeoxygenation processes is shown in **Figure** **7**.

The initial steps of the process involve the conversion of 2‐methoxy‐4‐propylphenol to 4‐propylphenol via hydrogen gas generated through the Raney Ni‐catalyzed dehydrogenation of isopropanol. The by‐products of this initial hydrogenolysis are acetone and methanol. Subsequently, 4‐propylphenol is hydrogenated to produce 4‐propylcyclohexanol, using a sufficient supply of hydrogen from isopropanol. This intermediate is then dehydrated over an HZSM‐5 catalyst to yield propylcyclohexene and water. Following this step, propylcyclohexene is hydrogenated with the aid of Raney Ni, leading to a high‐yield synthesis of propylcyclohexane. Additionally, through the cooperative catalysis of Raney Ni and HZSM‐5, propylcyclohexene is converted into propylbenzene with a yield of 62%. The water and methanol generated during the continuous reaction process undergo water‐phase reforming, facilitated by a bifunctional catalyst, producing a substantial amount of hydrogen gas.^[^ ^52^, ^53^ ^]^

While isopropanol serves as the primary hydrogen donor in the initial hydrogenation of 2‐methoxy‐4‐propylphenol to 4‐propylcyclohexanol, methanol and water function as auxiliary hydrogen sources during the subsequent hydrodeoxygenation steps to propylcyclohexane. This assertion is supported by comparative experiments detailed in the Supporting Information (Tables S5 and S6, Supporting Information). In experiments absent of methanol and water, using 4‐propylcyclohexanol as the substrate resulted in a 49.6% yield of propylcyclohexane (Table S5, Supporting Information). When methanol and water were present, the yield increased to 58.6%, affirming their role in in situ hydrogen generation via reforming reactions (Table S6, Supporting Information). These results verify that methanol and water are critical auxiliary hydrogen sources for the hydrodeoxygenation of 4‐propylcyclohexanol, while isopropanol remains the primary hydrogen donor. GC analysis confirmed the presence of acetone throughout the reaction, with a notable increase in its concentration at 200 °C in the presence of zeolite (Figure S9, Supporting Information). This observation validates the dehydrogenation of isopropanol as the primary pathway for H₂ generation.

For the production of methanol and water mentioned above, as well as whether the catalytic reforming reaction of methanol occurs in this system, experimental observations and GC testing were used to verify (Figure S10, Supporting Information). The blue anhydrous copper sulfate and headspace gas chromatography results indicate the presence of water and methanol, and a certain amount of CO_2_ and H_2_ in gas composition detection confirmed the occurrence of methanol catalytic reforming.

The reaction kinetics of 2‐methoxy‐4‐propylphenol, 4‐propylphenol, and 4‐propylcyclohexanol at different temperatures (Figures S11–S13, Supporting Information) provide a more detailed and comprehensive confirmation of the difficulty level of each reaction step in this system. First, the hydrogenolysis and intramolecular hydrogenation processes of 2‐methoxy‐4‐propylphenol are relatively rapid, completing the conversion within 1 h. Heating promotes this process to occur faster. Figure S11a (Supporting Information) shows that 4‐propylphenol exists in the form of an intermediate product, and the reaction kinetics of 4‐propylphenol at 140 °C (Figure S12a, Supporting Information) confirm that 4‐propylphenol can complete intramolecular hydrogenation to produce 4‐propylcyclohexanol at low temperatures. The heating process gradually promotes the cleavage of alcohol hydroxyl groups on 4‐propylcyclohexanol, and through dehydration and hydrogenation processes, more propylcyclohexane products are generated (Figure S12c, Supporting Information). Based on the relevant data of reaction kinetics, the activation energy of the main steps was calculated and fitted (Figure 7; Figure S14, Supporting Information). The hydrogenolysis of 2‐methoxy‐4‐propylphenol (127 KJ mol^−1^) and the intramolecular hydrogenation of 4‐propylphenol (137 KJ mol^−1^) were significantly easier than the hydrodeoxygenation of 4‐propylcyclohexanol (303 KJ mol^−1^).

For a more lucid elucidation of the conversion process, we have evaluated the product distribution at varying reaction durations under optimized conditions (Figure S15a, Supporting Information). As the procedure advanced at elevated temperatures, the acidic sites of HZSM‐5 became progressively active, augmenting the breakdown of the alcohol hydroxyl group in 4‐propylcyclohexanol. In conjunction with an ample supply of hydrogen, this facilitated the generation of more propylcyclohexane. Initially, 4‐propylphenol underwent hydrogenation to yield 4‐propylcyclohexanol. However, beyond 100 min, the deoxygenation of HZSM‐5 surpassed the hydrogenation of Raney Ni, resulting in an increased yield of propylbenzene. The contrasting production trends of 4‐propylcyclohexanol and propylcyclohexane highlighted that HZSM‐5 exhibited a more potent deoxygenation action in comparison to that on 2‐methoxy‐4‐propylphenol and 4‐propylphenol, indicating the efficient progression of the reaction.

Taking the reaction kinetics of 2‐methoxy‐4‐propylphenol at 200 °C as an example, the hydrogenation deoxygenation process is analyzed in detail. Based on the product distribution after different reaction times under optimized conditions, the reaction rate constant for each step is calculated (Figure S15b and Section S4, Supporting Information).^[^ ^54^ ^]^ The rate constant is expressed as k_1_ (9.90), indicating that the process of converting 2‐methoxy‐4‐propylphenol to 4‐propylphenol is very fast. The product distribution observed at the 1 min mark in Figure S15a (Supporting Information) during the initial stage of the reaction indicates that after the formation of 4‐propylphenol, it rapidly undergoes hydrogenation to form 4‐propylcyclohexanol, leaving only negligible unconverted 4‐propylphenol.

For a more definitive analysis of the conversion pathway of 4‐propylphenol, we have executed a supplemental experiment utilizing 4‐propylphenol as the initial reactant under identical reaction conditions, with the results display in Figure S12d and Table S7 (Supporting Information). Concurrently, we have computed the rate constants for the transformation of 4‐propylphenol to 4‐propylcyclohexanol and propylbenzene. The *k*_2_ (6.40) for the hydrogenation of 4‐propylphenol to 4‐propylcyclohexanol is notably higher than the *k*_3_ (1.02) for the deoxygenation from 4‐propylphenol to propylbenzene, indicating that in the early phase of hydrodeoxygenation, hydrogenation predominates, albeit with concomitant localized deoxygenation.

Subsequent catalytic deoxygenation of the resultant 4‐propylcyclohexanol through the Raney Ni&HZSM‐5 catalyst generates propylcyclohexane, as corroborated by the experiment with 4‐propylcyclohexanol as the substrate (Figure S13d and Table S5, Supporting Information). This experiment also reveals a minor dehydrogenation component, forming some propylbenzene, with the *k*_5_ (3.30) for the deoxygenation reaction greatly surpassing the *k*_4_ (1.93) for the dehydrogenation reaction. As the reaction proceeds, the acid function of HZSM‐5 becomes progressively active and eventually predominant, making the hydrogenation of subsequently formed propylbenzene to propylcyclohexane more challenging and resulting in a lower rate constant, *k*_6_ (0.02).

The analysis delineates a strategy to modulate the conversion of lignin monomer derivatives into desired products through hydrogenation and deoxygenation pathways. To preferentially synthesize alkane products, the hydrogenation reaction should initially be predominant, with subsequent deoxygenation reactions serving in a supportive role. This can be achieved by augmenting the hydrogen‐donating solvent or optimizing the experimental conditions, or by fine‐tuning the ratio of the Raney Ni&HZSM‐5 catalyst. Conversely, to favor the production of aromatic compounds, deoxygenation should be the principal reaction, aided by hydrogenation, perhaps by increasing the proportion of the HZSM‐5 catalyst.

**Figure 8:**
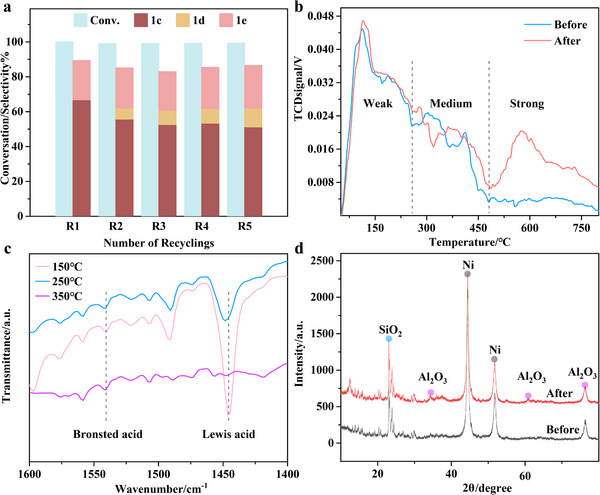
a) The yield distribution of products after each reaction of the Raney Ni&HZSM‐5 catalyst after 5 cycles of testing; b) NH_3_‐TPD; c) Pyridine adsorption Fourier‐transform infrared (Py‐IR); d) X‐ray Diffractometer (XRD). Reaction condition: 2 mmol 2‐methoxy‐4‐propylphenol (1a), 1.0 g Raney Ni, 0.25 g zeolite, 10 mL isopropanol, 200 °C, 240 min. After each reaction, filter, wash with isopropanol, dry at 60 °C, and then add to the reaction vessel. Add the substrate and 10 mL of isopropanol solvent again for the reaction. Abbreviations and full names in the picture: propylbenzene (1c), 4‐propylphenol (1d), propylcyclohexane (1e).

To evaluate the cost‐effectiveness of our approach, we assessed the catalyst's recyclability. Data presented in **Figure** **8**a indicate that the conversion rate of the substrate was not significantly affected even after five reaction cycles, and propylphenol gradually increased. Combined with NH_3_‐TPD spectrum analysis (Figure 8b), after 5 cycles, the strong acid sites of the Raney Ni&HZSM‐5 catalyst increased significantly. Due to the activation temperature of the strong acid sites being much higher than the reaction temperature, the more strong acid sites covering the surface hindered the cleavage effect of the weak acid sites on the phenolic hydroxyl groups of propylphenol, resulting in a decrease in the yield of propylbenzene and an increase in the content of propylphenol. By detecting the infrared spectra of pyridine in the temperature range of weak acid and medium strong acid (Figure 8c), there was a significant change in the pyridine adsorption intensity of the catalyst at 1450 cm^−1^ between 150 and 350 °C, indicating that the weak acid sites were mainly Lewis acid types. Comparing the content of different acid types before and after the reaction, the acidity of the catalyst increased after 5 cycles. The enhanced Al_2_O_3_ absorption peaks at 35° and 61° by XRD also confirm the claim of increased Bronsted acidity (Figure 8d), and the crystal structure of the zeolite did not significantly collapse or degrade before and after the reaction.

The stability and integrity of the Raney Ni&HZSM‐5 catalyst was assessed through electron microscopy mapping before and after its inaugural reaction.^[^ ^55^ ^]^ Interestingly, the catalyst demonstrated resilience with no apparent signs of structural degradation such as etching or collapse, and the elemental distribution remained uniform (Figure S16, Supporting Information), suggesting its recyclability.

The elucidated reaction mechanism and comprehensive experimental validation offer substantial theoretical backing and guidance for the industrial enhancement of lignin derivatives. Consequently, five distinct production pathways, based on different product requirements (Figure S17, Supporting Information), have been designed to yield 4‐propylcyclohexanol, propylcyclohexene, propylcyclohexane, propylbenzene, and a blend of propylcyclohexane and propylbenzene that can be directly utilized as alkane fuel. Route 1 employs Raney Ni as a catalyst, isopropanol as the hydrogen donor, and heat at 140 °C for 2 h to accomplish the hydrogenolysis and hydrogenation conversion of 2‐methoxy‐4‐propylphenol, resulting in a 92.2% yield of 4‐propylcyclohexanol. Acetone that produced through the dehydrogenation of isopropanol can be re‐hydrogenated to isopropanol under nickel catalysis, facilitating solvent recycling. Route 2 incorporates an additional step of HZSM‐5 catalytic deoxygenation reaction (200 °C, 2 h) onto the framework of Route 1 to attain a 93.3% yield of propylcyclohexene. Building upon Route 2, Route 3 further leverages Raney Ni for a distinct catalytic hydrogenation process, leading to a high yield of propylcyclohexane (93.2%). Route 4 extends Route 2 by using a Ni&HZSM‐5 bifunctional catalyst with a higher metal acid ratio (5:3, mass ratio) and heating at 200 °C for 4 h to obtain a 62% yield of propylbenzene. Route 5 directly employs the Ni&HZSM‐5 bifunctional catalyst to convert 2‐methoxy‐4‐propylphenol into a blend of propylcyclohexane and propylbenzene, featuring the same proportion as current commercial fuels and high purity. This mixture exclusively comprises C9 cycloalkanes and aromatics, serving as an essential fuel component. In comparison to prior investigations on the hydrogenation and deoxygenation of lignin‐derived monomers (Table S8, Supporting Information), this work showcases outstanding product diversity and controllability, realizing sustainable and efficient value‐added transformation in the absence of external hydrogen gas.

## 3 Conclusion

In this study, the utilization of commercial non‐precious metal catalysts and solid acid molecular sieves for a straightforward physical mixing process results in the formation of a bifunctional catalyst, Raney Ni&HZSM‐5, known for its efficacy in the transfer hydrodeoxygenation of lignin‐derived monomers into five valuable products: propylcyclohexane, suitable for alkane fuel applications, 4‐propylcyclohexanol and propylcyclohexene, utilized in organic synthesis, and propylbenzene, optimal for benzene‐based platform chemicals. Additionally, a mixture of propylcyclohexane and propylbenzene can serve as a direct jet fuel. Through the leveraging of the distinct response temperatures of metals and solid acids within bifunctional catalysts, as well as the high substrate sensitivity to the metal‐to‐acid ratio in these catalysts, five process flowcharts have been formulated. At suitable temperatures and catalyst ratios, we have achieved yields of 92.2% for 4‐propylcyclohexanol (Raney Ni, 140 °C), 93.3% for propylcyclohexene (HZSM‐5, 200 °C), 93.2% for propylcyclohexane (Raney Ni, 140 °C), 62.0% for propylbenzene (Raney Ni&HZSM‐5 mass ratio of 2:1), and 97.1% for alkane fuel (Raney Ni&HZSM‐5 mass ratio of 3:1), respectively. Notably, these transformations were achieved using the solvent hydrogen supply method under moderate conditions. Isopropanol acts as the primary hydrogen donor during the transfer‐hydrogenation and hydrodeoxygenation processes, whereas methanol and water play a secondary role as hydrogen sources in subsequent hydrodeoxygenation steps through in situ H₂ reforming. The validity of the reaction pathways was supported by the analysis of intermediates, revealing the roles of transfer hydrogenation by the Raney Ni and deoxygenation by the HZSM‐5. Moreover, the combined catalysts proved to be highly reusable and universally effective for the hydrodeoxygenation of various lignin monomer derivatives, establishing a versatile approach for the strategic conversion of lignin into valuable fuels and chemicals under gentle conditions. This advancement represents a promising step toward the resource‐efficient and controlled valorization of lignin.

## 4 Experimental Section

### Materials

2‐methoxy‐4‐propylphenol (1a), 4‐propylphenol (1d), propylbenzene (1c), palladium on carbon (Pd/C), and ruthenium on carbon (Ru/C) were obtained from Shanghai Aladdin Biochemical Technology Co., Ltd. Ni/SiO_2_‐Al_2_O_3_ was sourced from Alfa Aesar Chemical Co., Ltd. Methanol, ethanol, ethylene glycol, and glycerol were acquired from Tianjin Fuyu Fine Chemical Co., Ltd. Raney Ni and isopropanol were purchased from Shanghai Rhawn Chemical Technology Co., Ltd. 4‐propylcyclohexanol (1b) was sourced from Shanghai Bide Pharmaceutical Technology Co., Ltd. Zeolites, including HZSM‐5, H‐Y, H‐β, MOR, MCM‐41, and USY, were produced from Zhuoran Environmental Protection Technology Co., Ltd.

### Methods

A total of 2 mmol of lignin monomers was taken and mixed with specified quantity amount of catalyst. This was followed by the addition of 5–15 mL of isopropanol to the mixture. The resulting mixture was then transferred into a 25 mL high‐pressure reactor where it underwent a reaction at temperatures spanning 140–200 °C for a duration ranging from 0 to 4 h. Upon completion and subsequent cooling, the reaction mixture was subjected to filtration. A sample of the filtrate was diluted and placed into a GC vial for analysis. The final yield and distribution of the products were then determined by GC results.

### Characterizations

The product distribution was determined using an Agilent 8860 GC system equipped with an HP‐5 column and a flame ionization detector (FID). The GC thermal program involved initial retention at 40 °C for 5 min, a temperature ramp of 10 °C min^−1^ up to 265 °C, followed by a further increase to 300 °C at a rate of 15 °C min^−1^ with a final hold for 5 min. Substrate conversion and product selectivity were quantified utilizing the area normalization method.

NH_3_‐TPD analysis was performed using a temperature‐programmed chemical adsorption analyzer. A 50–100 mg sample of zeolite was positioned in a reaction tube and was progressively heated from ambient temperature to 500 °C at a rate of 10 °C min^−1^ for drying and pre‐treatment processes. Subsequently, the sample was purged with helium gas at a flow rate of 30–50 mL min^−1^ for 1 h. Upon cooling to 50 °C, the zeolite was exposed to a 10% NH_3_/He mixture at a flow rate of 30–50 mL min^−1^ for 1 h to achieve saturation. To eliminate NH_3_ weakly adsorbed on the zeolite surface, helium flow was resumed at a rate of 30–50 mL min^−1^ for an additional hour. The temperature was finally elevated to 800 °C at 10 °C min^−1^ under a helium atmosphere to facilitate desorption, while a thermal conductivity detector monitored the desorbed gases.

The BrukerTensor 27 pyridine infrared spectrometer was used to determine the acid type and relative acid content of the catalyst. Three temperature points (150, 250, 350 °C) were tested in a vacuum environment, with 32 infrared scans and a resolution of 4 cm^−1^.

XRD patterns were obtained using an X‐ray diffractometer with a Cu‐Kα radiation source operating at 40 kV and 40 mA. The zeolite sample was measured at a scanning rate of 5° min^−1^ within the 2*θ* range of 5° to 90°.

## Conflict of Interest

The authors declare no conflict of interest.

## Author Contributions

H.Y. and Y.Z.L. supervised the project. Y.Y. designed the experiments and carried out most of the experiments. Y.L.L., Y.H.L., and M.C. contributed to part of the experiments. Y.L.L. and Y.H.L. contributed to the results discussion. Y.Y., Y.Z.L., and H.Y. collectively wrote the paper. All authors commented on the final manuscript.

## Supporting information



Supporting Information

## Data Availability

The data that support the findings of this study are available in the supplementary material of this article.
