# Supplementary material for: Tunable Transfer‐Hydrodeoxygenated Upgrading of Lignin‐Derived Propylphenols to Versatile Value‐Added Alkane‐Based Chemicals
Source: Adv Sci (Weinh). 2025 Mar 11;12(17):2500687. doi: 10.1002/advs.202500687 (PMC12061236; doi:10.1002/advs.202500687)
Supplement: Supplementary file 1 — Supporting Information [file ADVS-12-2500687-s001.docx]

**Supporting information**

**Tunable Transfer-Hydrodeoxygenated Upgrading of Lignin-Derived Propylphenols to Versatile Value-added Alkane-based Chemicals**

Yanyan Yu, Yilin Li, Yuhan Lou, Mengyuan Chen, Yongzhuang Liu^*^, Haipeng Yu^*^

*Key Laboratory of Bio-based Material Science and Technology of Ministry of Education, Northeast Forestry University, Harbin 150040, PR China*

* Corresponding: yuhaipeng20000@nefu.edu.cn; lyz@nefu.edu.cn

**1. Characterization of zeolites**

1.1 The morphology and elemental distribution of Raney Ni&HZSM-5 before and after reaction were observed using the ZEISS Gemini 300 scanning electron microscope (SEM). A gold-palladium alloy gold-sprayed target was used, and the SEM operated at an accelerating voltage range of 0.02-15 kV. The magnification for the observations ranged from 10,000x to 100,000x.

1.2 The headspace gas chromatography (HS-GC) method was used to determine the presence of methanol in the liquid after the reaction. The instrument used was a Fuli 9790Plus+HS930 chromatograph, equipped with a TG-WAXMS chromatographic column and FID detector. An appropriate amount of sample was weighed, diluted with water, and 1mL was placed in a headspace bottle for machine testing. The injection port temperature was 150 ℃ and the detector temperature was 240 ℃. The column temperature was first maintained at 50 ℃ for 4 minutes, and then increased to 200 ℃ at a rate of 20 ℃ per minute. Headspace conditions: equilibrium temperature of 70 ℃, valve box temperature of 110 ℃, transmission line temperature of 115 ℃.

1.3 Collect the gas after the reaction using a gas collection bag, detect the gas composition after the reaction using a Thermo Fisher gas chromatograph, and equip TCD (chromatographic column model Mol Sieve 5A: 6ft × 1/8in × 2.1 mm SS and TG Packaged Column 2.0 m × 2.0 mm ID 1/8in OD) and FID (TG-BOND Aluminum [Na2SO4] 50 m × 0.32 mm × 5 um) detectors were used to detect H_2_ and alkane content, with an injection volume of 1mL. The temperature of the injection port is 80 ℃, the temperature of the TCD detector is 200 ℃, the temperature of the FID detector is 200 ℃, the split ratio is 14:1, the column flow rate is 2.5 ml/min, and the chromatographic column heating program is: maintain at 60 ℃ for 9.5 minutes, raise at 20 ℃ per minute to 200 ℃, and maintain for 2.5 minutes.

**2. Supporting Figures**

**Figure S1** Raney Ni catalyst exhibits substrate conversion and product selectivity at different temperatures. Reaction condition: 2 mmol substrate (1a), 1.0 g Raney Ni, 10 mL isopropanol, 140–200 ℃, 240 min. Abbreviations and full names in the picture: 2-methoxy-4-propylphenol (1a), 4-propylcyclohexanol (1b), propylbenzene (1c), 4-propylphenol (1d), propylcyclohexane (1e).

**Figure S2** The products distribution of the reaction at different temperatures when adding HZSM-5 zeolite. Reaction condition: 2 mmol substrate (1a), 1.0 g Raney Ni, 0.25 g HZSM-5, 10 mL isopropanol, 140–200 ℃, 240 min. Abbreviations and full names in the picture: 2-methoxy-4-propylphenol (1a), 4-propylcyclohexanol (1b), propylbenzene (1c), 4-propylphenol (1d), propylcyclohexane (1e).

**Figure S3** Digital photos of catalyst state after reaction at different temperatures. a) 140 ℃, b) 200 ℃. Reaction condition: 2 mmol 2-methoxy-4-propylphenol (1a), 1.0 g Raney Ni, 0.25g HZSM-5, 10 mL isopropanol, 240 min.

**Figure S4** Peak spectra of products in gas chromatography-mass spectrometry analysis under the condition of a 1:1 ratio of Raney Ni to HZSM-5. Reaction condition: 2 mmol 2-methoxy-4-propylphenol, 0.3 g Raney Ni, 0.3 g HZSM-5, 10 mL isopropanol, 200 ℃, 240 min.

**Figure S5** Standard substance position in GC. Abbreviations and full names in the picture: 2-methoxy-4-propylphenol (1a), 4-propylcyclohexanol (1b), propylbenzene (1c), 4-propylphenol (1d), propylcyclohexane (1e).

**Figure S6** Adding Raney Ni to different substrates to verify the hydrogenation effect of metal catalysts. Reaction condition: 2 mmol substrate (**①**: 2-methoxy-4-propylphenol; **②**: 4-propylphenol; **③**: propylbenzene), Raney Ni (1.0 g), 10 mL isopropanol, 200 ℃, 240 min.

**Figure S7** Verifying the deoxygenation effect of zeolite by adding HZSM-5 to different substrates (**④**: 2-methoxy-4-propylphenol; **⑤**: 4-propylphenol; **⑥**: 4-propylcyclohexanol). Reaction condition: 2 mmol substrate, HZSM-5 (0.25 g), 10 mL isopropanol, 200 ℃, 240 min.

**Figure S8** GC results of two-step hydrodeoxygenation of 4-propylcyclohexanol (1b) and synergistic catalysis of 1b by Raney Ni&HZSM-5 bifunctional catalyst. **⑦-1**: HZSM-5 (0.25 g), **⑦-2**: Ni (1 g), **⑧**: One-step addition of Raney Ni (0.5 g) and HZSM-5 (0.3 g) to verify the dehydrogenation pathway for the conversion of 4-propylcyclohexanol to propylbenzene. Reaction condition: 2 mmol 4-propylcyclohexanol, 10 mL isopropanol, 200 ℃, 240 min.

**
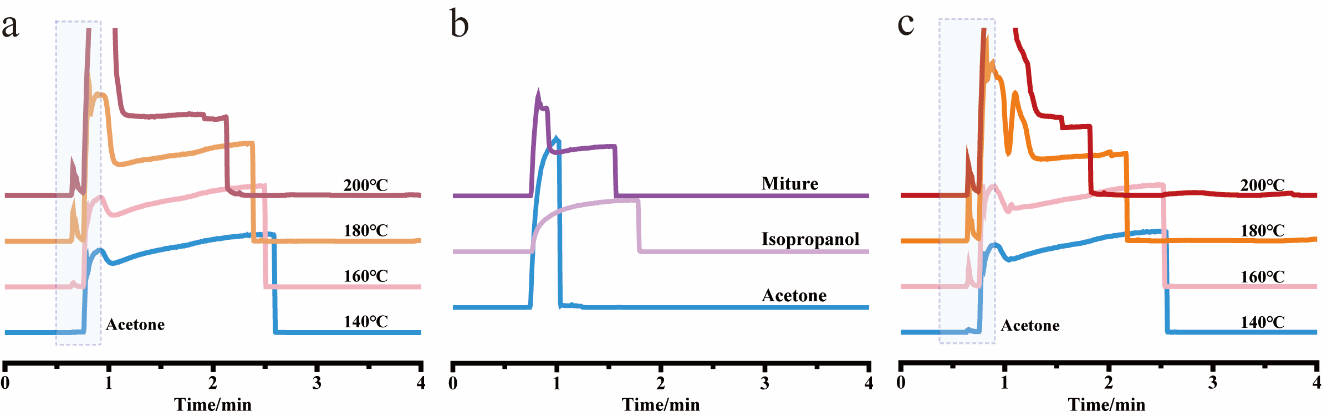
**

**Figure S9** Gas chromatography comparative detection of acetone products in hydrodeoxygenation reaction. a) The variation of acetone product with reaction temperature after hydrogenation deoxygenation of 2-methoxy-4-propylphenol without zeolite participation, b) Peak positions of acetone standard, isopropanol standard, and their mixtures in the gas chromatography, c) The variation of acetone product with reaction temperature in the presence of zeolite. Reaction condition: 2 mmol 2-methoxy-4-propylphenol, 1.0 g Raney Ni, 0.25 g zeolite (HZSM-5), 10 mL isopropanol, 200 ℃, 240 min.

**Figure S10** a) Detection of gas composition generated after hydrogenation deoxygenation reaction in 1a; b) The content of methanol in the solution after hydrogenation deoxygenation of 1a; c) Pressure changes in the container during the 1a hydrogenation deoxygenation process; d) The test for the formation of water on substrate 1a after hydrogenation deoxygenation at 140 ℃ and 200 ℃ shows that anhydrous copper sulfate turns blue, indicating the presence of water in the reaction solution, and white indicates the absence of water; e) Test for the generation of water after the reaction of propylcyclohexanol (1b) and propylbenzene (1c) separately. Reaction condition: 2 mmol 2-methoxy-4-propylphenol (1a), 1.0 g Raney Ni, 0.25 g zeolite, 10 mL isopropanol, 200 ℃, 240 min; ①Reaction temperature: 200 ℃, ②Reaction temperature: 140 ℃.


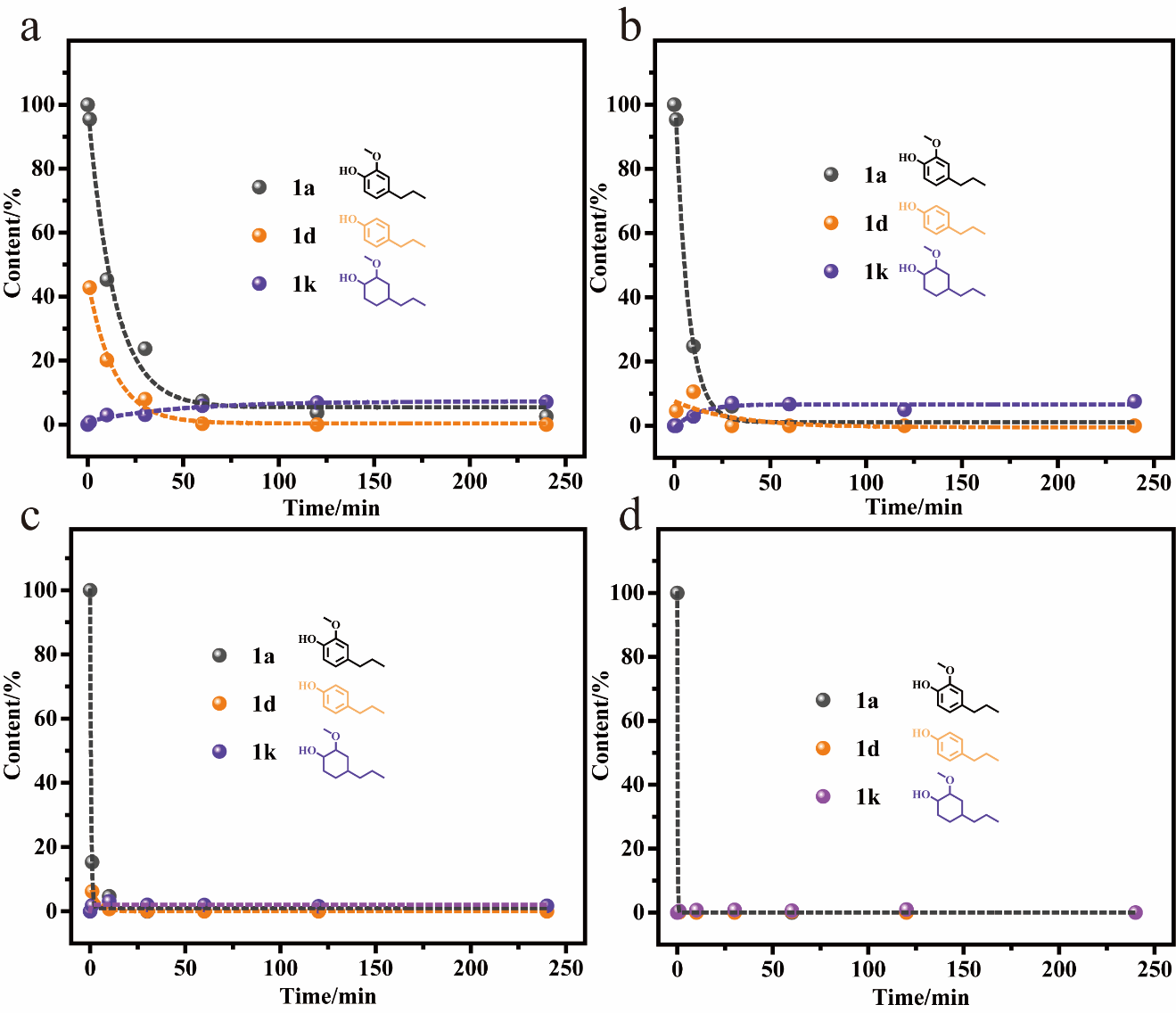


**Figure S11** Content variation curves of 2-methoxy-4-propylphenol (1a) and main products with different reaction times. a) 140 ℃, b) 160 ℃, c) 180 ℃, d) 200 ℃. Reaction condition: 2 mmol substrate, 1.0 g Raney Ni, 0.25 g zeolite, 10 mL isopropanol, 140 ℃, 1 min, 10 min, 30 min, 60 min, 120 min, 240 min. Abbreviations and full names in the picture: 4-propylphenol (1d), 2-methoxy-4-propylcyclohexane (1k).


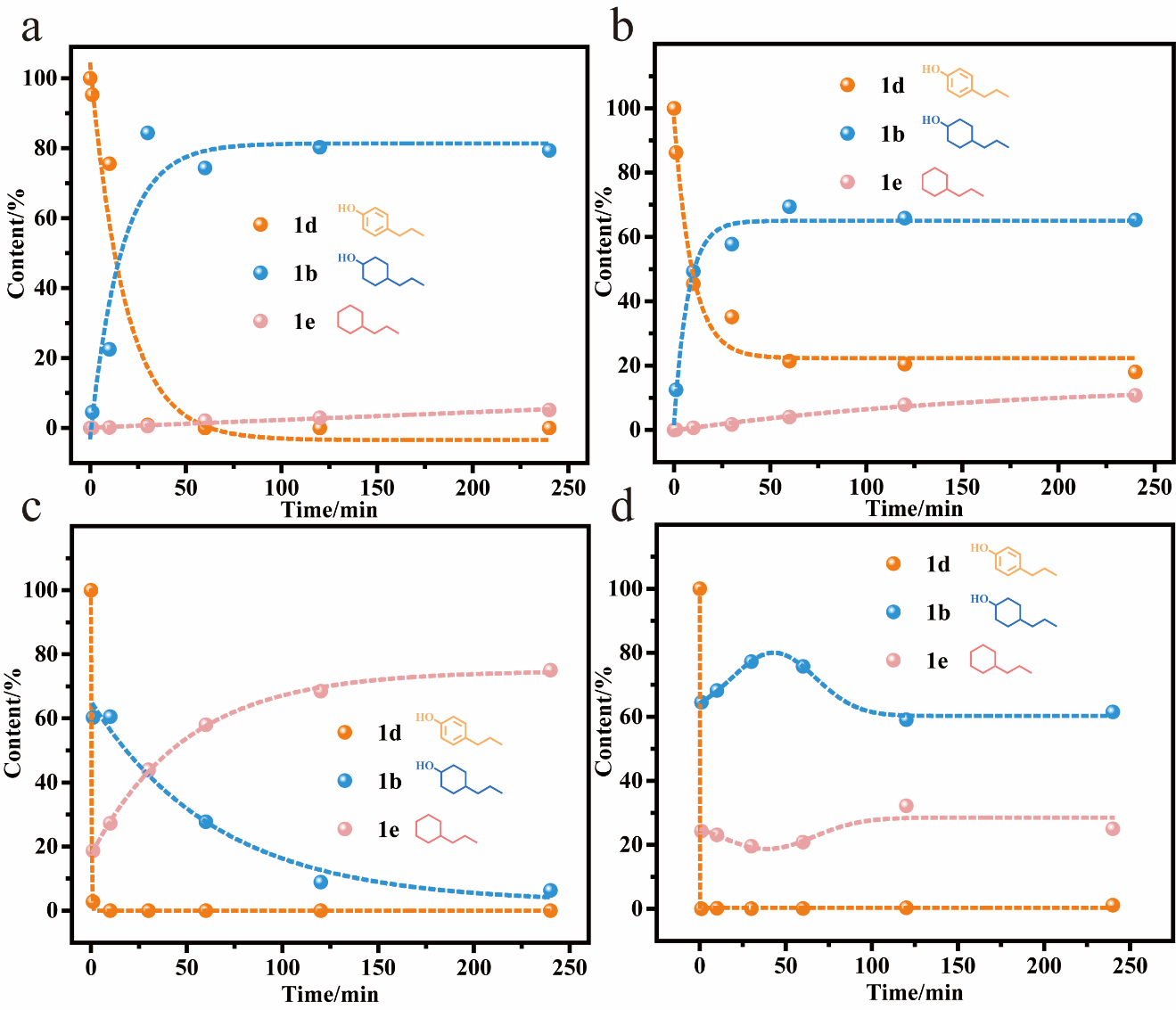


**Figure S12** Content variation curves of 4-propylphenol (1d) and main products with different reaction times. a) 140 ℃, b) 160 ℃, c) 180 ℃, d) 200 ℃. Reaction condition: 2 mmol substrate, 1.0 g Raney Ni, 0.25 g zeolite, 10 mL isopropanol, 140 ℃, 1 min, 10 min, 30 min, 60 min, 120 min, 240 min. Abbreviations and full names in the picture: 4-propylcyclohexanol (1b), propylcyclohexane (1e).


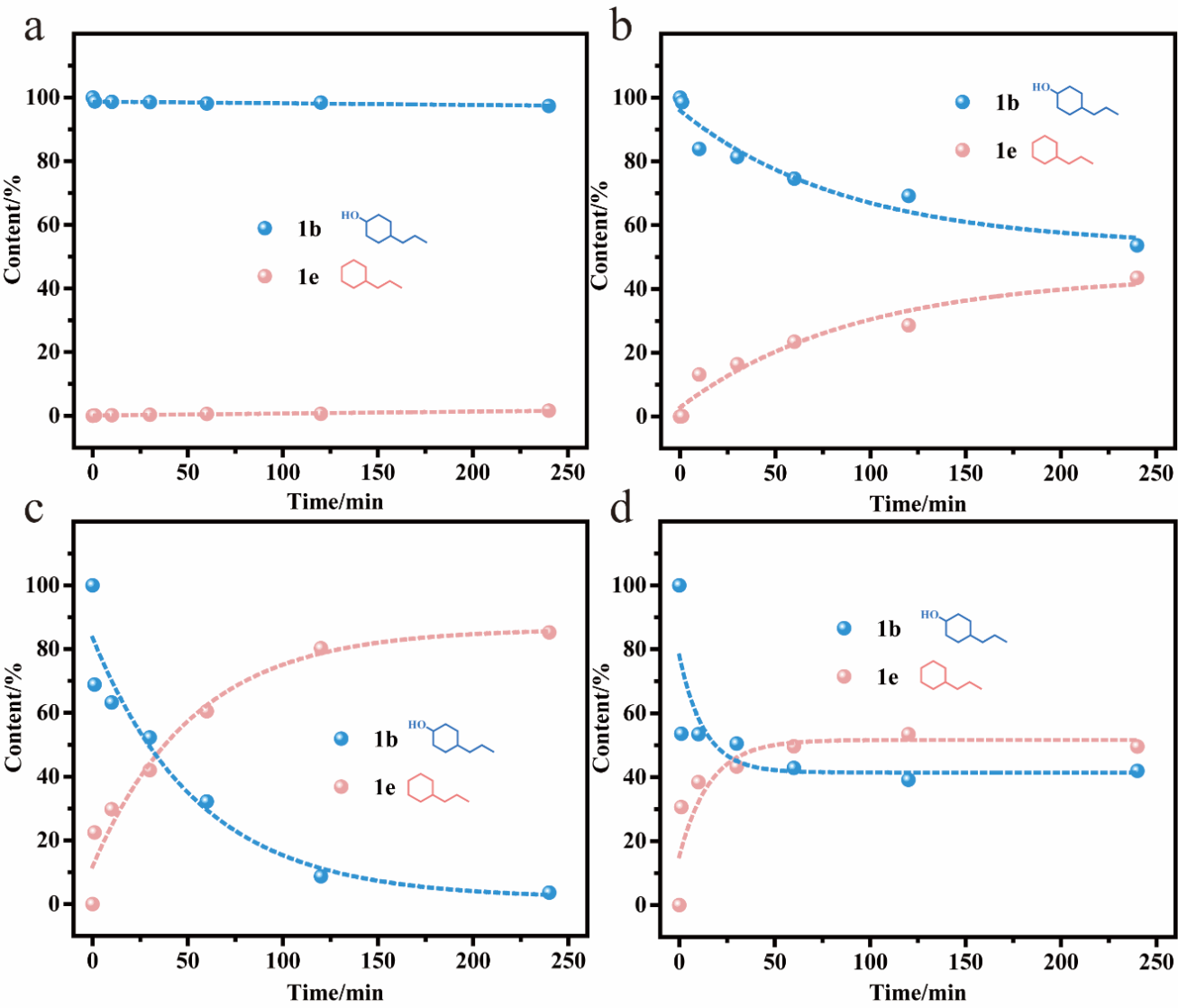


**Figure S13** Content variation curves of substrate 4-propylcyclohexanol (1b) and main product (propylcyclohexane, 1e) with different reaction times. a) 140 ℃, b) 160 ℃, c) 180 ℃, d) 200 ℃. Reaction condition: 2 mmol substrate, 1.0 g Raney Ni, 0.25 g zeolite, 10 mL isopropanol, 140 ℃, 1 min, 10 min, 30 min, 60 min, 120 min, 240 min.

**
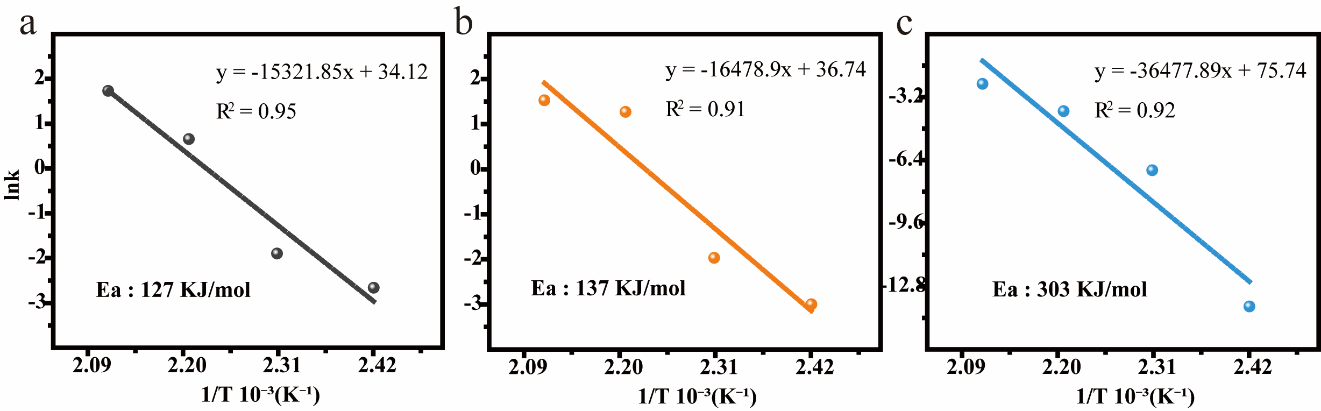
**

**Figure S14** Curve fitting of reaction activation energy for a) 2-methoxy-4-propylphenol, b) 4-propylphenol, c) 4-propylcyclohexanol.

**Figure S15** a) The yield of each product under different reaction time; b) Reaction kinetic path diagram for 2-methoxy-4-propylphenol. Reaction condition: 2 mmol 2-methoxy-4-propylphenol, 1.0 g Raney Ni, 0.25 g zeolite, 10 mL isopropanol, 200 ℃, 0–240 min. Abbreviations and full names in the picture: 2-methoxy-4-propylphenol (1a), 4-propylcyclohexanol (1b), propylbenzene (1c), 4-propylphenol (1d), propylcyclohexane (1e).


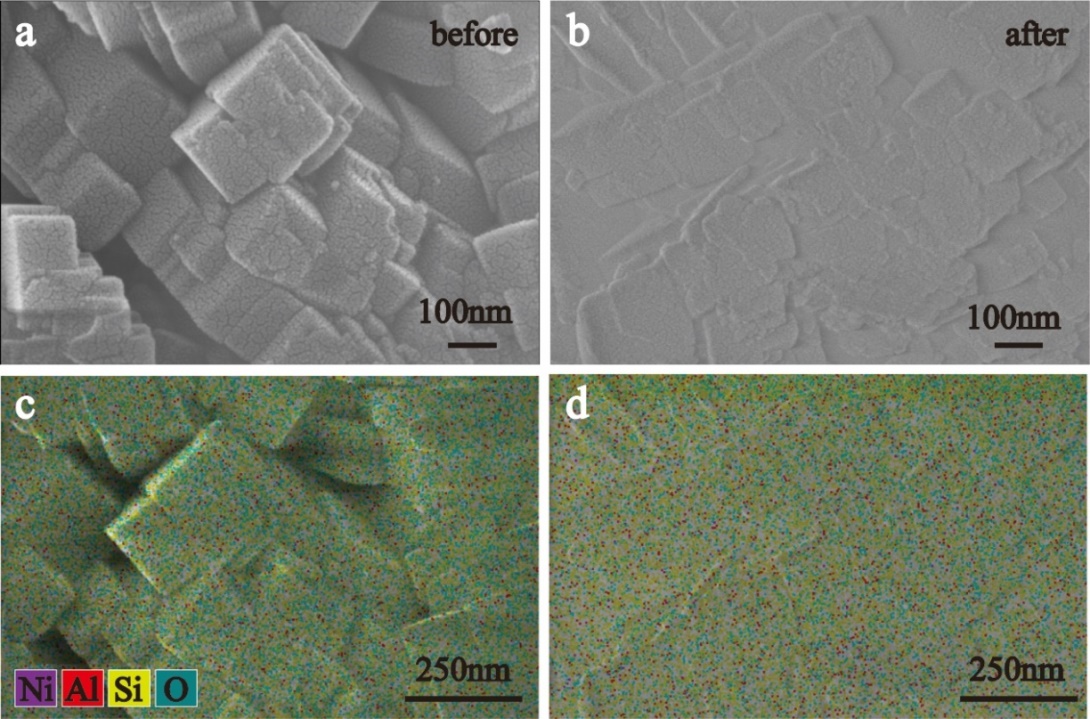


**Figure S16** Scanning electron microscopy mapping of Raney Ni&HZSM-5 before and after reaction.


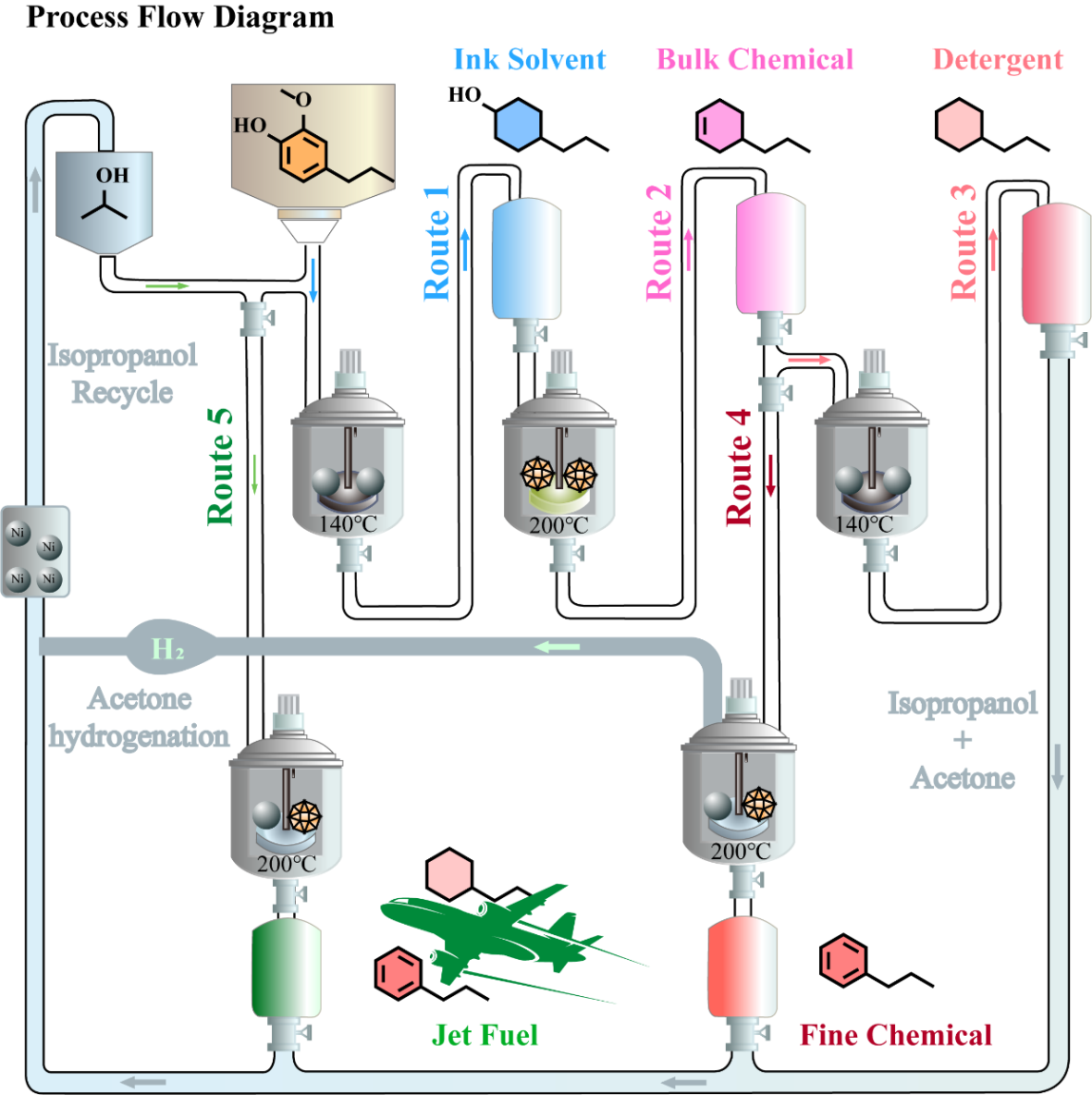


**Figure S17** Production process diagram of different high-value chemicals.

**3. Supporting Tables**

**Table S1** NH_3_ adsorption and desorption test results of three zeolites and bifunctional catalyst.

| catalyst | Total acid sites (mmol NH_3_/g) | Acid sites (mmol/g) | |
| --- | --- | --- | --- |
|  |  | week | strong |
| HZSM-5 | 7.16 | 5.69 | 1.47 |
| H-β | 9.54 | 9.17 | 0.37 |
| H-Y | 18.79 | 18.79 | 0 |
| Raney Ni&HZSM-5 | 0.52 | 0.31 | 0.21 |

**Table S2** Detection of gas composition after hydrodeoxygenation reaction.

| Gas type | C_3_H_8_ | CH_4_ | CO_2_ | H_2_ |
| --- | --- | --- | --- | --- |
| Content% | 81.29 | 9.98 | 3.11 | 2.36 |

Reaction condition: 2 mmol substrate (2-methoxy-4-propylphenol), 1.0 g Raney Ni, 0.25 g zeolite, 10 mL isopropanol, 200 ℃, 240 min.

**Table S3** Headspace gas phase analysis of methanol generation after reaction.

| Reaction temperature (℃) | Reaction time (min) | Methanol content (%) |
| --- | --- | --- |
| 140 | 1 | 0.16 |
| 140 | 240 | 0.56 |

Reaction condition: 2 mmol 2-methoxy-4-propylphenol, 1.0 g Raney Ni, 0.25 g zeolite, 10 mL isopropanol, 140 ℃, 1 min or 240 min.

**Table S4** Pyridine infrared spectroscopy results of Raney Ni&HZSM-5 before and after reaction.

| Pyridine adsorption temperature (℃) | Bronsted acid content (umol/g) | Lewis acid content (umol/g) | Total acid content (umol/g) | B/L |
| --- | --- | --- | --- | --- |
| 150^a^ | 0.37 | 8.36 | 8.73 | 0.04 |
| 150^b^ | 0.48 | 13.04 | 13.52 | 0.04 |
| 250^a^ | 0.26 | 3.88 | 4.14 | 0.07 |
| 250^b^ | 0.37 | 4.84 | 5.21 | 0.08 |
| 350^a^ | 0.16 | 0.20 | 0.36 | 0.79 |
| 350^b^ | 0.26 | 0.48 | 0.74 | 0.55 |

a: Fresh catalyst, b: Catalyst after 5 cycles of reaction. Reaction condition: 2 mmol 2-methoxy-4-propylphenol, 1.0 g Raney Ni, 0.25 g zeolite, 10 mL isopropanol, 200 ℃, 240 min.

**Table S5** Conversion of 4-propylcyclohexanol (1b) and yield of each product under different reaction times.

| Time/min | Conversion %  (1b) | Yields % | | | |
| --- | --- | --- | --- | --- | --- |
|  |  | 1e | 1c | 1d | 1f |
| 1 | 46.41 | 30.64 | 13.89 | 0.05 | 0.82 |
| 10 | 46.53 | 38.43 | 7.02 | 0.03 | 0.29 |
| 30 | 49.48 | 43.34 | 4.75 | 0 | 0.63 |
| 60 | 57.11 | 49.65 | 5.5 | 0 | 0.94 |
| 120 | 60.8 | 53.44 | 4.71 | 0.09 | 1.21 |
| 240 | 58.02 | 49.59 | 5.06 | 0.23 | 1.46 |

Reaction condition: 2 mmol 4-propylcyclohexanol, 1.0 g Raney Ni, 0.25g zeolite, 10 mL isopropanol, 200℃, 0–240 min. Abbreviations and full names in the table: propylbenzene (1c), 4-propylphenol (1d), propylcyclohexane (1e), propylcyclohexene (1f).

**Table S6** Conversion of 2-methoxy-4-propylphenol (1a) and yield of each product under different reaction times.

| Time/min | Conversion %  (1a) | Yields % | | | | |
| --- | --- | --- | --- | --- | --- | --- |
|  |  | 1e | 1c | 1b | 1d | 1f |
| 1 | 99.99 | 19.48 | 10.52 | 67.69 | 1.60 | 0.25 |
| 10 | 100 | 30.98 | 6.99 | 61.08 | 0 | 0.17 |
| 60 | 100 | 36.12 | 2.74 | 59.01 | 1.02 | 0.48 |
| 90 | 100 | 43.88 | 4.38 | 48.79 | 1.19 | 0.90 |
| 120 | 100 | 48.67 | 8.04 | 40.07 | 0.62 | 1.63 |
| 240 | 100 | 58.61 | 13 | 21.84 | 1.07 | 2.61 |

Reaction condition: 2 mmol 2-methoxy-4-propylphenol, 1.0 g Raney Ni, 0.25 g zeolite, 10 mL isopropanol, 200 ℃, 0–240 min. Abbreviations and full names in the picture: 4-propylcyclohexanol (1b), propylbenzene (1c), 4-propylphenol (1d), propylcyclohexane (1e), propylcyclohexene (1f).

**Table S7** Conversion of 4-propylphenol (1d) and yield of each product under different reaction times.

| Time/min | Conversion %  (1d) | Yields % | | | |
| --- | --- | --- | --- | --- | --- |
|  |  | 1e | 1c | 1b | 1f |
| 1 | 99.94 | 24.27 | 10.15 | 64.54 | 0.50 |
| 10 | 99.82 | 23.09 | 7.47 | 68.21 | 0.40 |
| 30 | 99.92 | 19.59 | 2.02 | 77.22 | 0.38 |
| 60 | 99.90 | 20.84 | 1.96 | 75.75 | 0.54 |
| 120 | 99.67 | 32.18 | 5.55 | 59.09 | 1.70 |
| 240 | 98.85 | 25.02 | 7.9 | 61.5 | 1.91 |

Reaction condition: 2 mmol 4-propylphenol, 1.0 g Raney Ni, 0.25 g zeolite, 10 mL isopropanol, 200 ℃, 0–240 min. Abbreviations and full names in the picture: 4-propylcyclohexanol (1b), propylbenzene (1c), propylcyclohexane (1e), propylcyclohexene (1f).

**Table S8** Comparison of reaction conditions and yields between this work and other works.

| Catalysts | Substrate | Temperature and Time | Gaseous hydrogen  (MPa) | Solvent | Conv.  (%) | Product  (Yield%) | Ref. |
| --- | --- | --- | --- | --- | --- | --- | --- |
| Raney Ni | 2-methoxy-4-propylphenol | 140℃, 4h | 0 | Isopropanol | 100 | Propylcyclohexanol (92.2) | This work |
| Raney Ni/HZSM-5 | 2-methoxy-4-propylphenol | 200℃, 4h | 0 | Isopropanol | 100 | Propylcyclohexene (93.3) | This work |
| Raney Ni/HZSM-5 | 2-methoxy-4-propylphenol | 200℃, 4h | 0 | Isopropanol | 100 | Propylcyclohexane (93.2) | This work |
| Raney Ni/HZSM-5 | 2-methoxy-4-propylphenol | 200℃, 4h | 0 | Isopropanol | 100 | Propylene (62.0) | This work |
| Ni/HZSM-5 | 2-methoxy-4- propylphenol | 250℃, 2h | 5 | H_2_O | 98 | Cycloalkanes (84.0) | [1] |
| Ni/Nafion/SiO_2_ | 2-methoxy-4- propylphenol | 300℃, 2h | 4 | H_2_O | 95 | Propylcyclohexane (73) | [2] |
| RuNPs@SILP-1.00 | 2-methoxy-4-methylphenol | 175℃, 16h | 12 | Decalin | >99 | Methylcyclohexane (77) | [3] |
| Ru/HZSM-5 | Guaiacol | 240℃, 4h | 0.2 (0.6 MPa N_2_) | H_2_O | 100 | Methylbenzene (90) | [4] |
| Ni/ZSM-5 | Guaiacol | 160℃, 24h | 3 | n-Hexane | 100 | Cyclohexane (89) | [5] |
| 10Ni-15W/NiAl_2_O_4_ | 2-methoxy-4-propylphenol | 250℃, 3h | 5 | Dodecane | 100 | Propylcyclohexane (93.5) | [6] |
| Ni1/β-Mo_2_C | 2-methoxy-4-propylphenol | 260℃, 2h | 4 | n-Undecane | 100 | Propylcyclohexanol (92.2) | [7] |
| Pd/C, HZSM-5 | 2-methoxyphenol | 240℃, 2h | 4 | H_2_O | 77.4 | Cyclohexane (5.9) | [8] |
| Pd/C | 2-methoxy-4-propylphenol | 250℃, 0.5h | 5 | H_3_PO_4_-H_2_O pH=2.1 | 100 | Propylcyclohexane (66) | [9] |
| Cu1Si1 | Toluene | 230℃, 6h | 5 | n-Dodecane | 12.5 | Methylcyclohexane (10.8) | [10] |
| PA-Cu/CuPMO | Bio-oil | 300℃, 8h | 0 | Methanol | -- | Cycloalkanes (10.04) | [11] |

**Table S9** List of abbreviations.

| Abbreviations | Full names |
| --- | --- |
| 1a | 2-methoxy-4-propylphenol |
| 1b | 4-propylcyclohexanol |
| 1c | propylbenzene |
| 1d | 4-propylphenol |
| 1e | propylcyclohexane |
| 1f | propylcyclohexene |
| 1i | 4-propanoxypropylbenzene |
| 1j | 3-Methoxy-4-propanoxypropylbenzene |
| 1k | 2-methoxy-4-propylcyclohexane |

**4. Reaction kinetic calculation**

4.1 The kinetic reaction formula for continuous reactions:

$\frac{ⅆC1a}{ⅆt}$= -k_1_C_1a_

$\frac{ⅆC1d}{ⅆt}$= k_1_C_1a_ – (k_2_+k_3_)C_1d_

$\frac{ⅆC1b}{ⅆt}$= k_2_C_1d_ – (k_4_+ k_5_)C_1b_

$\frac{ⅆC1c}{ⅆt}$= k_3_C_1d_ +k_4_C_1b_ – k_6_C_1c_

$\frac{ⅆC1e}{ⅆt}$= k_5_C_1b_ +k_6_C_1c_

By substituting the reaction product data for different time periods and performing a fitting calculation, the rate constants k for each reaction are as follows:

k_1_= 9.90

k_2_= 6.40

k_3_= 1.02

k_4_= 1.93

k_5_= 3.30

k_6_= 0.02

The fitted equations are as follows:

C_1a_= 0.2*exp(-9.9t)

C_1b_= 1.0/(0.02+7.42*t+5.23*t^2^)

C_1c_= 13.63-0.22*(exp(-1.93t)-exp(-0.02t))

C_1d_= 0.2*exp(-7.42t)

C_1e_= 22.99-0.074*exp(-5.23t)

4.2 The Arrhenius equation reflects the effect of temperature on the rate constant of chemical reactions, and the formula is as follows:

k=Ae^(-Ea/RT)^

Take the logarithm on both sides, and the formula becomes:

lnk = - (Ea/R)(1/T) + lnA

In the formula, k is the reaction rate constant; T represents absolute temperature; A represents the frequency factor; R is the molar gas constant, R=8.314J·mol^-1^·K^-1^; Ea is called activation energy. Ea can be calculated based on the slope of the fitted curve.

**References**

[1] C. Zhao, J. A. Lercher, *Angew. Chem. Int. Ed.* **2012**, *51*, 5935-5940. DOI: 10.1002/anie.201108306.

[2] C. Zhao, Y. Kou, A. A. Lemonidou, X. B. Li, J. A. Lercher, *Chem. Commun.* **2010**, *46*, 412-414. DOI: 10.1039/b916822b.

[3] K. L. Luska, P. Migowski, S. E. Sayed, W. Leitner, *Angew. Chem. Int. Ed.* **2015**, *54*, 15750-15755. DOI: 10.1002/anie.201508513.

[4] Z. X. Zheng, Z. C. Luo, C. Zhao, *ChemCatChem* **2018**, *10*, 1376-1384. DOI: 10.1002/cctc.20170.

[5] Y. Hu, X. M. Li, M. Y. Liu, S. Bartling, H. Lund, P. J. Dyson, M. Beller, R. V. Jagadeesh, *ACS Sustainable Chem. Eng.* **2023**, *11*, 15302-15314. DOI: 10.1021/acssuschemeng.3c03661.

[6] X. Zhang, J. F. Wu, T. Li, C. Z. Zhang, L. J. Zhu, S. R. Wang, *Chem. Eng. J.* **2022**, *429*, 132181. DOI: 10.1016/j.cej.2021.132181.

[7] H. Q. Guo, J. W. Zhao, Y. Chen, X. Y. Lu, Y. Yang, C. R. Ding, L. Z. Wu, L. Tan, J. L. Long, G. H. Yang, N. Tsubaki, X. L. Gu, *ACS Catal.* **2024**, *14*, 703-717. DOI: 10.1021/acscatal.3c04555.

[8] C. Zhang, J. B. Qi, J. Xing, S. F. Tang, L. Song, Y. Y. Sun, C. H. Zhang, H. C. Xin, X. B. Li, *RCS Adv.* **2016**, *6*, 104398. DOI: 10.1039/c6ra22492j.

[9] C. Zhao, J. Y. He, A. A. Lemonidou, X. B. Li, J. A. Lercher, *J. Catal.* **2011**, *280*, 8-16. DOI: 10.1016/j.jcat.2011.02.001.

[10] H. Wang, W. R. Zhao, M. U. Rehman, W. Liu, Y. X. Xu, H. J. Huang, S. P. Wang, Y. J. Zhao, D. H. Mei, X. B. Ma, *ACS Catal.* **2022**, *12*, 4724-4736. DOI: 10.1021/acscatal.2c00380.

[11] X. C. Kong, C. Liu, Y. Y. Fan, M. Li, R. Xiao, *ACS Sustainable Chem. Eng.* **2023**, *11*, 7454-7465. DOI: 10.1021/acssuschemeng.3c00426
